# An Extended Polyanion Activation Surface in Insulin Degrading Enzyme

**DOI:** 10.1371/journal.pone.0133114

**Published:** 2015-07-17

**Authors:** Eun Suk Song, Mehmet Ozbil, Tingting Zhang, Michael Sheetz, David Lee, Danny Tran, Sheng Li, Rajeev Prabhakar, Louis B. Hersh, David W. Rodgers

**Affiliations:** 1 Department of Molecular and Cellular Biochemistry and the Center for Structural Biology, University of Kentucky, Lexington, Kentucky, United States of America; 2 Department of Chemistry, University of Miami, Miami, Florida, United States of America; 3 Center for Computational Sciences, University of Kentucky, Lexington, Kentucky, United States of America; 4 Department of Medicine, University of California, San Diego, La Jolla, California, United States of America; Russian Academy of Sciences, Institute for Biological Instrumentation, RUSSIAN FEDERATION

## Abstract

Insulin degrading enzyme (IDE) is believed to be the major enzyme that metabolizes insulin and has been implicated in the degradation of a number of other bioactive peptides, including amyloid beta peptide (Aβ), glucagon, amylin, and atrial natriuretic peptide. IDE is activated toward some substrates by both peptides and polyanions/anions, possibly representing an important control mechanism and a potential therapeutic target. A binding site for the polyanion ATP has previously been defined crystallographically, but mutagenesis studies suggest that other polyanion binding modes likely exist on the same extended surface that forms one wall of the substrate-binding chamber. Here we use a computational approach to define three potential ATP binding sites and mutagenesis and kinetic studies to confirm the relevance of these sites. Mutations were made at four positively charged residues (Arg 429, Arg 431, Arg 847, Lys 898) within the polyanion-binding region, converting them to polar or hydrophobic residues. We find that mutations in all three ATP binding sites strongly decrease the degree of activation by ATP and can lower basal activity and cooperativity. Computational analysis suggests conformational changes that result from polyanion binding as well as from mutating residues involved in polyanion binding. These findings indicate the presence of multiple polyanion binding modes and suggest the anion-binding surface plays an important conformational role in controlling IDE activity.

## Introduction

Insulin degrading enzyme (IDE^3^, E.C. 3.4.24.16) is a zinc metallopeptidase widely expressed in mammalian cells. IDE is primarily localized to the cytosol, with lower amounts found in peroxisomes [1], endosomes [2], and mitochondria [3], and small amounts secreted from cells [4]. The substrate specificity of IDE is broad and not well defined. Although it has been suggested that the enzyme shows a preference for amyloidogenic substrates [5], there are many exceptions. IDE is one of the major Aβ-degrading enzymes in the brain and is a key enzyme involved in insulin catabolism. IDE also degrades glucagon, amylin, and atrial natriuretic peptide. The enzyme has been linked to both Alzheimer’s disease and Type 2 diabetes [6,7]. Recent studies implicate IDE as the key regulator of calcitonin gene–related peptide levels [8]. Other roles for IDE include regulating proteasome activity [9] and acting as a non-catalytic chaperone-like activity upon amyloid-forming peptides [10]. Recently Tundo et al [11] reported that IDE is stress inducible acting like a heat shock protein and regulating cell growth.

Structural studies of IDE show that it is a homodimer [12] with each monomeric unit composed of four structural domains [13]. When the kinetics of the enzyme was examined using the small synthetic substrate Abz-GGFLRKHGQ-EDDnp, IDE displayed allosteric kinetics [12]. This kinetic behavior was attributable to a second molecule of substrate binding to an extended part of the substrate-binding site that we named the distal site [14]. In addition, we found that IDE activity could be modulated by polyanions including triphosphate and nucleotide triphosphates including free ATP [15]. We showed that mutations that disrupt polyanion binding reduced the activity of IDE in yeast [16], showing that the polyanion-binding site is functional in yeast and likely in mammalian cells as well.

We were able to crystallize IDE with ATP bound and identified the region of the enzyme to which it binds [16]. However the properties of the IDE-ATP complex suggested that there were other binding modes for ATP. In the present study, we employ a combined molecular docking and all-atom molecular dynamics simulations approach to identify potential ATP binding modes inside the substrate-binding cavity of IDE. Mutagenesis was used to characterize the kinetic consequences of disrupting each of these binding modes. Molecular dynamics and hydrogen-deuterium exchange were used to assess possible conformational changes that could lead to affects on activity.

## Materials and Methods

### Identification of ATP binding sites by computational analysis

We employed a multistep computational strategy to investigate the interactions of ATP with IDE. In the first step, the X-ray structure (PDB ID: 3P7L) of substrate-free rat IDE was equilibrated using all-atom 30 ns molecular dynamics (MD) simulations in an aqueous solution using the GROMACS program [17,18] and the GROMOS96 53A6 force field [19]. In the simulations, the starting structures were placed in a large cubic box (12.0 x 12.0 x 12.0 nm^3^) to avoid artificial interactions with their images in the neighboring boxes created by the application of periodic boundary conditions. The box was filled with single point charge (SPC) water molecules. Some water molecules were replaced with sodium and chloride ions (160 Na^+^ and 134–138 Cl^-^) to neutralize these systems and to simulate an experimentally used ion concentration of 150 mM. The starting structures were subsequently energy-minimized with a steepest descent method for 3000 steps. The results of these minimizations produced the starting structure for the MD simulations. The MD simulations were then carried out with a constant number of particles (N), pressure (P) and temperature (T) i.e. NPT ensemble. The SETTLE algorithm was used to constrain the bond length and angle of the water molecules [20], while the LINCS algorithm was used to constrain the bond length of the protein [21]. The long-range electrostatic interactions were calculated by the Particle-Mesh Ewald (PME) method [22]. A constant pressure of 1 bar was applied with a coupling constant of 1.0 ps peptide, water molecules and ions were coupled separately to a bath at 300 K with a coupling constant of 0.1 ps. The equation of motion was integrated at each 2 fs time steps using leap-frog algorithm [23]. The tools available in the GROMACS program package and the YASARA software (v.13.2.2) were utilized for analyzing trajectories and simulated structures [24]. The most representative structure provided by these simulations was subsequently used to study IDE-ATP interactions. The most representative structures were derived from cluster analysis, where the trajectories were analyzed by grouping structurally similar frames (root-mean-square deviation cutoff = 0.30 nm) [25], and the frame with the largest number of neighbors was denoted as a middle structure that represented that particular cluster.

In the next step, an ATP molecule (taken from PDB ID: 2W74) was docked inside the cavity of IDE using the Autodock Vina 1.1.2 software. [26]. This procedure was performed using two different grid assignments. In the first assignment, a large grid box that encompassed the entire cavity of IDE was used, while in the second, thirteen small grid boxes were utilized to cover the whole cavity. This analysis ensured that the potential binding sites in the whole cavity were explored. In these docking experiments the protein was kept rigid, but the ATP molecule had the flexibility to adopt different conformations. The result provided 350 poses. Based on binding energies and the composition of interacting sites, the most promising poses were selected to perform short-term 5 ns MD simulations. These simulations checked the stability of docking sites and allowed ATP to move into a more favorable conformation. In the next step, the most promising poses provided by the docking procedure were subjected to 30 ns all-atom MD simulations in an aqueous solution. As above, these simulations were performed using GROMACS [17,18] and GROMOS96 53A6 [19]. MD simulations of several point mutant enzyme forms were also carried by first making the point mutations in COOT [27] and then carrying out all atom simulations in aqueous solution as described above for the wild type enzyme. The secondary structure analyses were performed by employing the defined secondary structures of proteins (DSSP) protocol [28]. The RMSD of all trajectories indicated that they were well equilibrated within the 30 ns time frame (**S1 and S2 Figs**).

### Preparation of IDE mutants

IDE mutants were generated using the QuikChange mutagenesis kit (Stratagene) with rat IDE (residues 42–1019) as the template. The following primers were used:

R429S forward, 5’-TTT AAA GAT AAA GAG AGC CCA CGA GGC TAC ACA-3’

reverse, 5’-TGT GTA GCC TCG TGG GCT CTC TTT ATC TTT AAA-3’

R431A forward, 5’-GAT AAA GAG AGG CCA GCA GGC TAC ACA TCT AAG-3’

reverse, 5’-CTT AGA TGT GTA GCC TGC TGG CCT CTC TTT ATC-3’

R847T forward, 5'—GGC ATC CAG GGC TTG ACA TTC ATC ATC CAG TCA-3'

reverse, 5'-TGA CTG GAT GAT GAA TGT CAA GCC CTG GAT GCC-3'

K898A forward, 5’-CGA CTC GAC AAA CCA GCG AAA CTC TCT GCA GAG-3’

reverse, 5'-CTC TGC AGA GAG TTT CGC TGG TTT GTC GAG TCG-3'

### Expression and purification of IDE mutants

IDE and its mutant forms were expressed in Sf9 cells as hexahistidine fusion proteins with a linker containing a tobacco etch virus protease cleavage site and purified on a His-select nickel affinity gel (Sigma) column. The purity of each IDE form was judged to be greater than 90% by SDS-PAGE. Protein concentration was determined using the Coomassie Blue reagent with BSA as the standard.

### Enzyme activity assays

For routine activity assays we used the fluorogenic peptide Abz-GGFLRKHGQ-EDDnp as previously described (24). Reaction mixtures contained 10 μM peptide in 50 mM Tris- HCl, pH 7.4, IDE, and when added ATP in a total volume of 200 μl. The reaction was monitored at 37°C for 30 min. using a SpectraMax Gemini XS fluorescence plate reader.

### Kinetic analysis

Kinetic data were fit to either a hyperbolic substrate versus velocity response curve (v = V_max_ + [S]/Km + [S]) or to a sigmoidal response curve (v = (V_max_ [S]^H^/(Km^H^ + [S]^H^), where H is the Hill coefficient and S is the substrate concentration) using GraphPad software. The K_A_ for ATP binding and the maximal fold activation were determined by fitting the data to the equation v^relative^ = B_max_ [ATP]/(K_A_ + [ATP]) +1, where v^relative^ is the observed fold rate increase in the presence of a given concentration of ATP, B_max_ is the maximal fold increase in reaction rate at saturating ATP, and K_A_ is the concentration of ATP that produces 50% maximal activation. Kinetic experiments were performed two or three times with different enzyme preparations.

### Hydrogen-deuterium exchange with mass spectrometry (DXMS)

Before conducting DXMS experiments, the optimal quench and pepsin proteolysis conditions were obtained as previously described [29] to maximize the peptide sequence coverage of IDE. A protein digestion solution (12 μl), prepared by mixing 3 μl stock solution of IDE (18 μM) with 9 μl of water, was quenched on ice with 18 μl of 16.6% glycerol, 0.8% formic acid containing various concentrations (0.08, 0.8, 1.6 and 3.2 M) of guanidine hydrochloride (GuHCl) and then frozen on dry ice. The quenched, frozen samples were later subjected to a DXMS protocol for analysis (see below). With 1.6 M GuHCl in the quench solution, we obtained 98% coverage of IDE, and this condition was used for subsequent deuteration studies.

For DXMS, all exchange stock solutions contained 12.7 μM IDE, 8.3 mM Tris, 50 mM NaCl, pH 7.2, with or without 50 mM ATP. This mixture was incubated at room temperature for 15 min and then kept at 0°C. Deuterium exchange studies were initiated by mixing 3 μl of exchange stock solution (IDE or IDE-ATP) with 9 μl of D_2_O buffer (8.3 mM Tris, 50 mM NaCl, pD_read_ 7.2) and incubating for varying times (10, 100, 1,000, 10,000, and 100,000 sec) at 0°C. The exchange reaction was quenched by the addition of 18 μl of pre-cooled optimal quench buffer (1.6 M GuHCl, 0.8% formic acid, 16.6% glycerol) at 0°C, and the samples were immediately frozen on dry ice. Un-deuterated control samples and back exchange control samples (incubated in D_2_O buffer containing 0.8% formic acid for 24 hr at 25°C) were also prepared.

The frozen samples were thawed automatically at 4°C using a modified AS3000 autosampler and injected onto a solid-phase porcine pepsin column for on-line digestion. The digested peptides were then directed to a C18 trap column for desalting. A linear acetonitrile gradient (6.4–38.4% over 30 min at 2 μl/min) was applied to a Michrom C18 column (MAGIC C18AQ 0.2x50 mm) to separate resulting peptides and the eluate introduced into an OrbiTrap Elite mass spectrometer (ThermoFisher Scientific, San Jose, CA) for MS analysis. To identify the likely sequence of the parent peptide ions, Proteome Discoverer software was used to perform protein database searching with the acquired MS/MS data. The extent of deuterium incorporation was determined using DXMS Explorer software (Sierra Analytics, LLC, Modesto, CA), which calculates centroid values automatically. Correction for back-exchange was employed using the method of Zhang & Smith [30,31].

## Results

### Docking and molecular dynamics simulations

**Fig 1:**
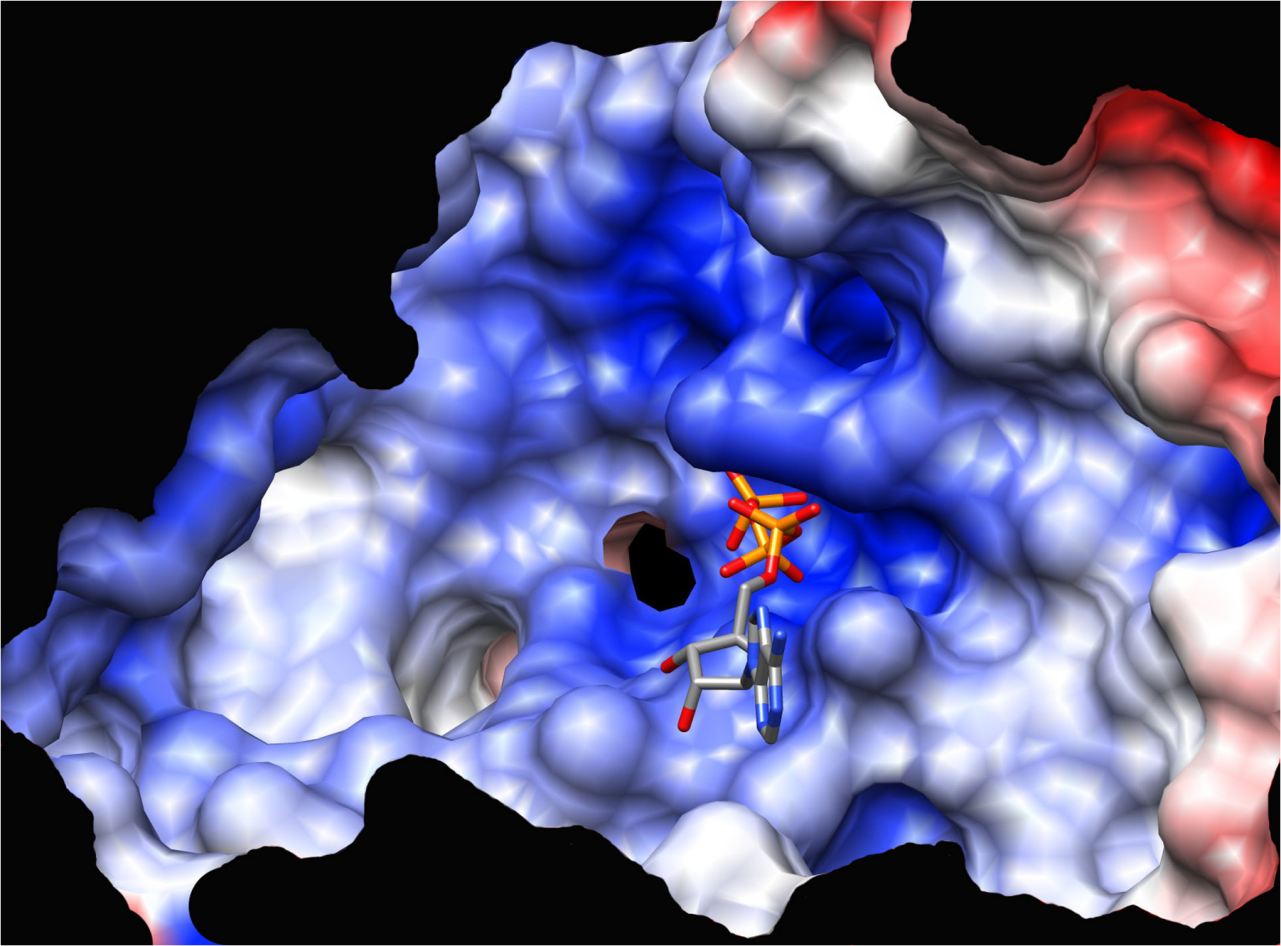
Polyanion binding surface. The molecular surface responsible for polyanion binding shown with surface electrostatic potential indicated by blue (positive) and red (negative) coloring (a +/- 10 kT cutoff used). The position of bound ATP in the IDE-ATP complex crystal structure (16) is indicated.

We previously determined the crystal structure of IDE with the polyanion ATP bound (16). This structure was used to identify the IDE allosteric activation site that binds polyanions and increases IDE activity more than 50 fold toward the synthetic substrate Abz-GGFLRKHGQ-EDDnp. ATP was used as a model polyanion and was shown to activate IDE only in its metal-free state with little if any contribution from the adenosine moiety [15]. This polyanion binding site lies within a highly cationic region between domains 3 and 4 (**Fig 1**). Using site-directed mutagenesis coupled with kinetic analyses we previously confirmed that the crystallographically identified polyanion-binding site was indeed an allosteric activation site [16]. However, this analysis suggested that within this highly charged cationic surface there were likely alternative modes of ATP binding.

**Fig 2:**
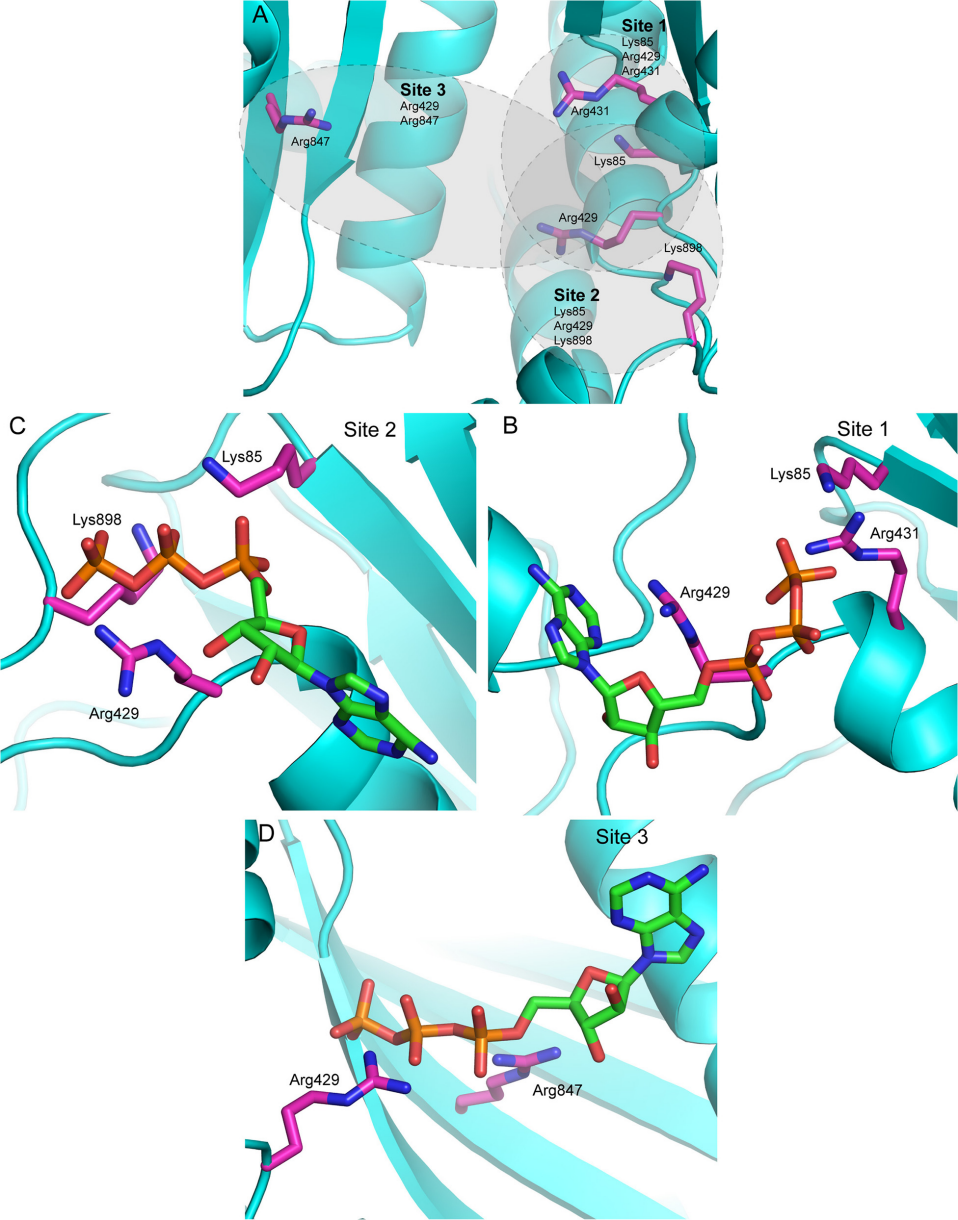
Three computationally identified ATP binding sites. (A) The three ATP binding sites found by docking and molecular dynamics are indicated by gray shading. The view is similar to that shown in **Fig 1**. Site 2 corresponds to the ATP binding site found in the IDE-ATP complex crystal structure (16) as shown in **Fig 1**. Side chains for residues mutated in the work reported here are indicated by stick figures. (B-D) Close up views of the three binding sites showing final ATP positions from the molecular dynamics simulations and the side chains of basic residues involved in binding. The superposition of structures at 5, 10, 15, 20, 25, and 30 ns indicated that ATP is bound to site 1 and 2 throughout the simulation, while it moved to site 3 only in the last 10 ns of the simulation, suggesting the initial position of the ligand was not a high affinity site (**S3–S5 Figs**).

We now have utilized a combined computational approach and experimental site-directed mutagenesis approach to identify the alternative ATP binding modes within the cationic region. These docking and MD simulations produced three potential ATP binding sites (**Fig 2**). In these sites, ATP was found to be stable with overall low root mean square deviation (RMSD) values (< 1.0 Å on all ligand atoms) throughout the simulations. In site 1, Arg 429 and Arg 431 are putative polyphosphate interacting residues, while in site 2 Arg 429, Lys 898, interact with the polyphosphate moiety. In site 3, Arg 429 and Arg 847 interact with the polyphosphate moiety of ATP. Site 2 is the location originally identified by crystallography as an ATP binding site, while sites 1 and 3 are new sites implicated by the computational studies.

**Fig 3:**
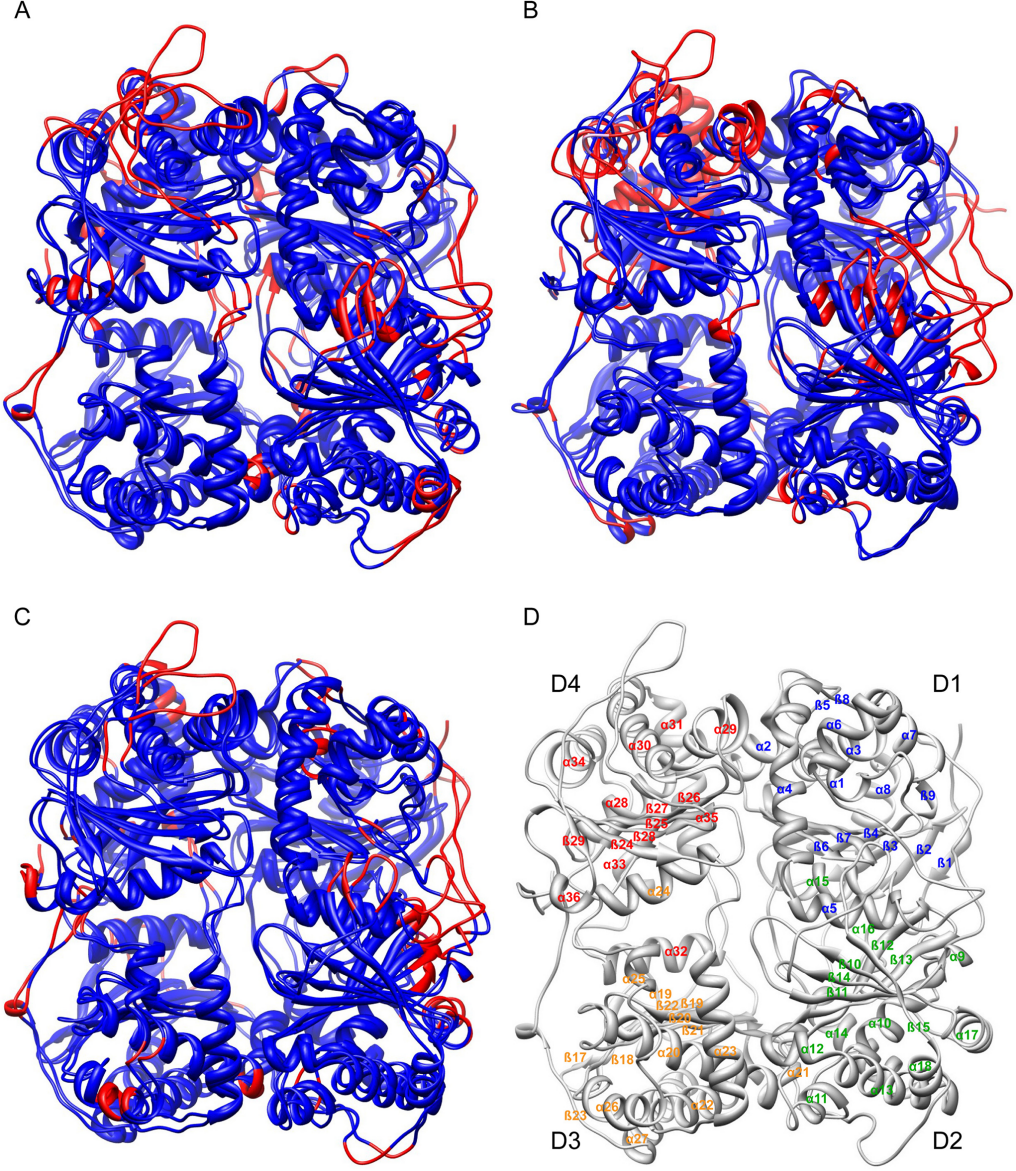
Dynamic simulations of unliganded and ATP bound IDE structures. Superpositions of representative structures from molecular dynamics simulations of unliganded IDE and IDE with ATP bound at (A) site 1, (B) site 2, or (C) site 3. Differences between Cα positions greater than 3.0 Å are highlighted in red for both of the superimposed ribbons representations.

A DSSP analysis showed only small changes in the secondary structure of ATP-bound IDE relative to unliganded IDE (**S6–S9 Figs; Table 1)**, a finding in agreement with the crystallographic structure of the ATP-IDE complex. The calculated overall RMSDs of ATP-bound IDE relative to ligand-free IDE were 2.7 Å, 2.8 Å, and 2.7 Å on all Cα positions for ATP bound in polyanion sites 1, 2, and 3 respectively. Interestingly, a number of structural elements at the half molecule interface alter conformation relative to the unliganded enzyme (**Fig 3**). In particular, elements in the hinge region (α11, α21) at the interface between domains 2 and 3 alter conformation when ATP is bound at any of the three sites. Shifts are also found, somewhat more variably depending on ATP-binding mode, in other interface elements, including portions of helices α2, α4, α5, α11, α14, α21, α28, α29 and α31, and the coil segments connecting helices α14 and α15, α23 and α24 and α31 and α32 (**Table 2**). These changes at the interface between halves of the molecule likely affect the strength of the interaction between the two IDE domains and, as a consequence, the partitioning between the open and closed forms of the molecule. Outside the interface region, a number of other structural elements in the ATP-bound simulations show changes relative to the unliganded enzyme. In domain 2, which includes the distal allosteric site, strands of the central sheet as well as helices α17 and α18 shift in all three simulations, which is consistent with the connection established between the ATP binding surface and the distal site [16,32]. In addition, the helix bearing catalytic residues, α1, shifts in the site 2 and site 3 simulations, and the last helical element, α36 and the C-terminal strand, β30, which form part of the dimer interface, differ in the site 1 and site 3 simulations. It is likely that alterations to catalytic residues the affect activity of the enzyme, and changes to the C-terminal elements may alter the dimer interface. It is known that dimerization is required for allosteric activation [33], and a change to the interface may be part of this allosteric mechanism. In the site 3 simulation, elements of the domain 4 central sheet and all of the core helices shift relative to the unliganded enzyme, a more extensive change than seen for the other two polyanion sites. Finally, a number of the extended loops also vary between all of the simulations, presumably due to their dynamic nature.

**Table 1:** Predicted Secondary Structure of IDE with ATP bound to its putative binding sites.

Secondary Structure	WT-IDE	ATP bound to Site 1	ATP bound to Site 2	ATP bound to Site 3
α-helix %	39.9	40.1	39.9	39.6
β-sheet %	19.5	20.6	19.2	21.5

**Table 2:** Magnitude of shifts in selected structural elements between three ATP bound (sites 1, 2, or 3) IDE structures and unliganded IDE.

	Difference from Unliganded IDE^1^					
	Site 1		Site 2		Site 3	
	RMSD (Å)	residues	RMSD (Å)	residues	RMSD (Å)	residues
hinge region	3.2	350–352 421–422 605–609	3.9	351–352 522–529	3.1	352 605–608
half molecule interface	3.6	154–157 420–425 428–430 673–676	3.6	174–207 424–425 535–543 633–634 673–675 803–828 877–898	3.1	175–177 341–343 875–898
domain 2	3.9	200–205 317–318 466–470 487–499 508–519	3.2	316–318 362–363 368–370 462–467	3.4	316–320 365–371 460–471
active site helix	——	——	3.2	112–113	4.0	111–121
C-terminal elements	3.2	993–1011	——	——	3.1	997–1002

^1^ For elements that shift more than 3.0 Å based on Cα positions in each of the indicated regions (excluding some loops).

At a more detailed level, the interaction of ATP with the enzyme induces noticeable structural changes in the amino acid residues at all three binding sites and the active site. In the binding site 1, Arg431 rotates and Lys85 shifts to interact with the phosphate groups of the ligand (**S14 Fig**). Arg429 and Lys899 also shift to form hydrogen bonds with bound ATP. In the binding site 2, Arg429 shifts away from the binding site to accommodate the bound ATP (**S15 Fig**), as do turn residues 894–900. Phe141 rotates to accept a hydrogen bond aromatic interaction (NH-π) from the exocyclic amino group of adenine. Tyr150 also adjusts position to donate a hydrogen bond to N1 of the adenine. In binding site 3, the backbone around Arg839 shifts about 2.5 Å from WT-IDE to allow a hydrogen bond from the main chain nitrogen to the γ phosphate of ATP (**S16 Fig**). The side chain of Arg847 also shifts toward the ligand to make an interaction with the α phosphate. Met683 rotates away from the site to accommodate the bound ligand.

### Hydrogen-deuterium exchange with mass spectrometry

**Fig 4:**
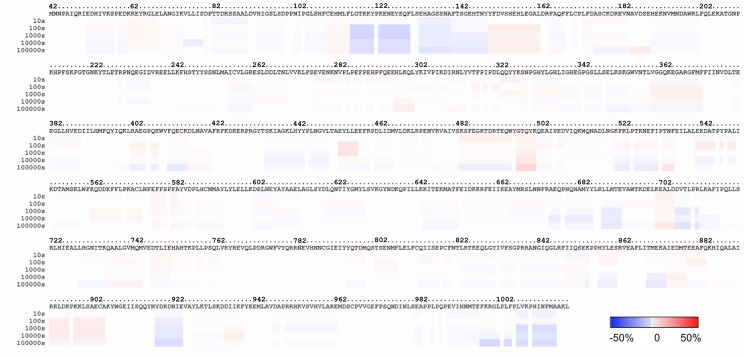
Hydrogen-deuterium exchange results for unliganded and ATP-bound IDE. A heat map of differences in exchange for unliganded IDE and IDE in the presence of 50 mM ATP is shown. Regions with slower exchange are shaded blue, and regions with more rapid exchange are shaded red, with the extent of changes indicated by the key.

**Fig 5:**
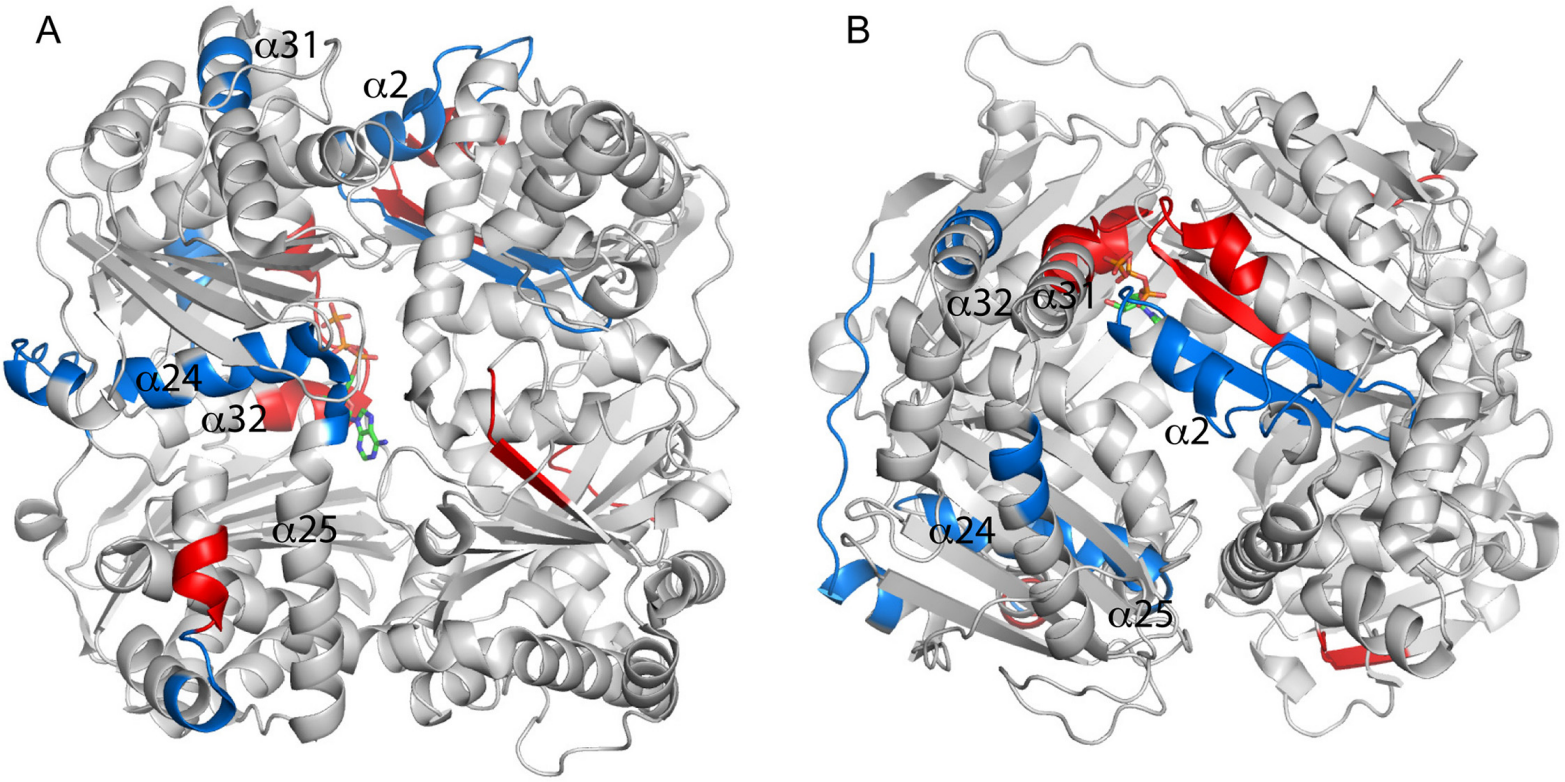
Structural elements with accessibility changes upon ATP binding. Regions of IDE with slower (blue) or more rapid (red) deuteration rate in the presence of ATP are shown in a ribbons representation of the protein. Secondary elements discussed in the text are labeled. The representations in panels A and B differ by a rotation of 90° about the horizontal axis.

Differential hydrogen-deuterium exchange studies show that binding of ATP causes changes in solvent accessibility for a number of structural elements (**Figs 4 and 5**), which include α24, the N terminus of α25, the C terminus of α31 and the N terminus of α32. Significantly, these elements flank the ATP binding sites that were defined by simulations and crystallography, supporting their role in polyanion binding. The affected regions of α24 and α25 become less accessible to proton exchange upon ATP binding (decrease of 17% at 1000 sec, residues 675–692), a frequently observed consequence of ligand interaction. The change in α31 and α32 is however toward increased exchange (increase of 5% at 1000 sec, residues 892–905). This effect also has been observed for ligand binding and has been proposed to result from an increased population of excited states, which mediate exchange, despite stabilization of the ground state by ligand interaction [34]. Interestingly, one of the other elements that alters exchange rate is helix α2 (decrease of 31% at 1000 sec, residues 127–133), which forms a key region of the interface between the two halves of the enzyme. This helix is one of the interface elements seen to alter conformation in a simulation of ATP binding, and the exchange result provides experimental verification of this effect. The change in C-terminal elements toward decreased exchange upon ATP binding (decrease of 11% at 1000 sec, residues 998–1016) is an intriguing result, since as noted above, the C-terminal residues form much of the dimer interface, and this result suggests that the dimer is stabilized by polyanion (ATP) binding.

### Site-directed mutagenesis

We used site-directed mutagenesis as a way to provide additional evidence that the three putative ATP binding sites actually contribute to activation of IDE by polyanions. The strategy employed involved mutating individual residues in each of the three putative polyanion binding sites as well as Arg429, which interacts with ATP in all three sites, and determining their effect on the activity of free IDE and the ability of ATP to increase IDE activity towards the substrate Abz-GGFLRKHGQ-EDDnp. Guided by our crystal structure of IDE, we chose substitutions that were predicted to be the least disruptive.

The results of the mutational analysis of the putative ATP binding sites are given in **Table 3**. Wild type IDE activity is maximally stimulated over 80 fold by 5 mM ATP, consistent with previously reported values [15], and all of the point mutations reduced the level of activation by 9–12 fold, strongly supporting the three proposed polyanion binding modes. In addition, mutations at sites 1 and 2 reduced the Hill coefficients to levels that indicate loss of substrate cooperativity. In terms of the basal activity of IDE, it is seen that mutations from each of the individual putative ATP binding sites modestly decreased basal activity, with the largest changes seen for the site 1 and 3 mutations (the maximum decrease in V_max_ of ~ 2.5 fold was found for the site 1 mutation R431A). These results suggest that there are likely conformational changes that occur when the targeted sites are mutated and that these conformational changes reduce the activity of unliganded IDE.

**Table 3:** Effect of mutating Putative Polyanion Binding Residues on IDE Activity and Activation by ATP.

IDE	V_max_ nmols/min/mg	K_m_ (μM)	Hill Coefficient	Fold Activation
wild type	32.2. ± 1.8	18.7 ± 3.4	1.6 ± 0.4	85.5 ± 6.7
**Site 1 Mutation**				
R431A	12.5 ±1.3	10.7 ± 2.5	1.2 ± 0.2	6.7 ± 0.4
**Site 2 Mutation**				
K898A	25.1 ± 3.1	20.6 ± 4.6	1.1 ± 0.08	7.4 ± 0.5
**Site 3 Mutation**				
R847T	16.0 ± 1.0	10.0 ± 1.3	1.4 ± 0.2	8.6 ± 0.4
**Site1, 2, 3 Mutation**				
R429S	24.7 ± 1.7	19.3 ± 3.2	1.5 ± 0.2	8.9 ± 0.2

**Fig 6:**
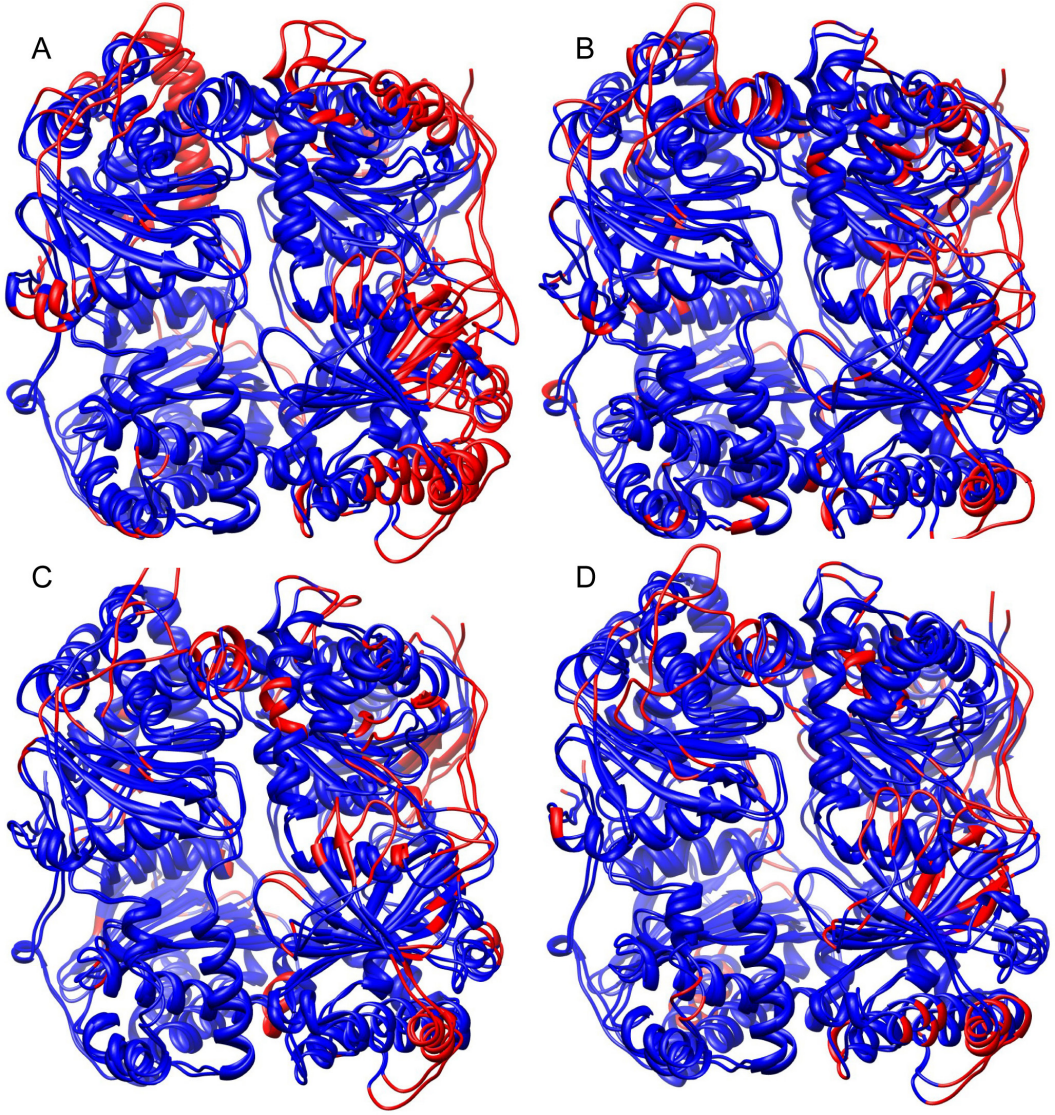
Dynamic simulations of wild type and mutant IDE structures. Representative IDE wild type and mutant structures from extended molecular dynamics simulations are superimposed with differences between Cα positions greater than 3.0 Å highlighted in red. (A) IDE^R431A^ (site 1 mutant) versus IDE^wt^. (B) IDE^K898A^ (site 2 mutant) versus IDE^wt^. (C) IDE^R847T^ (site 3 mutant) versus IDE^wt^. (D) IDE^R429S^ (all sites) versus IDE^wt^.

In order to provide structural insight into the changes in basal activity (see Table 2), we evaluated the mutant constructs by MD simulations. Four constructs were simulated: the site 1 mutant IDE^R431A^, the site 2 mutant IDE^K898A^, the site 3 mutant IDE^R847T^, and a mutant affecting all sites IDE^R429S^. As for the IDE-ATP simulations, DSSP analysis showed only small changes in the secondary structure of the IDE mutants relative to the wild type enzyme (**S6 Fig and S10–S13 Figs**). Interestingly, the site 1 mutant IDE^R431A^, which has the largest reduction in basal activity, shows the most extensive changes in the simulation relative to the wild type enzyme (RMSD on all Cα positions of 2.8 Å). Conformational changes observed in the simulations (**Fig 6; Table 4**) include a number of elements of domain 2 (central β-sheet elements and helices 11, 13, 17, 18), which contains the distal activation site. These alterations may account for the change in V_max_ as well as the reduction in Hill coefficient. At the site of the mutation, R431 interacts with the side chains of Asp152 and Asn139 from the central sheet of domain 1 in the wild type enzyme, and this interaction is absent in IDE^R431A^ (**S17 Fig**). Loss of this interaction likely causes the shift of helix α15 along is axis relative to the central sheet.

**Table 4:** Magnitude of shifts in selected structural elements between four point mutants (in ATP binding sites 1, 2, 3, or all) IDE structures and wild type IDE.

	Difference from Wild Type IDE^1^							
	R431A (Site 1)		K898A (Site 2)		R847T (Site 3)		R429S (all sites)	
	RMSD (Å)	residues	RMSD (Å)	residues	RMSD (Å)	residues	RMSD (Å)	residues
hinge region	3.9	342–343 351–353 424–425 534–538	3.1	351–353 525–529 655–658	3.1	343 606–607	4.2	350–352 526
half molecule interface	4.2	81–82 119–125 135–136 154–156 176–180 673–674 875–898	3.8	132–133 815–818	3.1	156 184–187 305–307 537–540 817–828 898–900	3.6	81–84 137 156–157 566–567 817–822 896–897
domain 2	4.3	295–300 316–320 365–373 388–406 462–477 491–498 508–521	4.4	204–207 210–216 298–299 486–488 492–497 508–521	3.4	318 462–466 484 491–498 504–521	3.9	317–326 351–352 362–367 388–392 483–488 492–499 518–520
active site helix	3.1	111–113	3.4	112–116	4.0	104–110	3.3	109–116
C-terminal elements	3.6	993–997 1007–1011	3.1	996-1000-1007	——	——	3.2	1001–1002

^1^ For elements that shift more than 3.0 Å based on Cα positions in each of the indicated regions (excluding some loops).

The IDE^R847T^ mutant (site 3) also shows a substantial reduction in V_max_ but differs from IDE^R431A^ in that it retains substrate activation. The MD simulation with this mutant shows less extensive changes (RMSD 2.4 Å) from IDE^wt^ than IDE^R431A^, and in particular changes to domain 2 are reduced, potentially accounting for retention of substrate cooperativity. The IDE^R847T^ simulation has the largest changes in the α1 helix bearing the catalytic residues, and conformational changes in the catalytic machinery may account for the reduction in V_max_ in this mutant. Arg847 in the wild type enzyme is located in the central sheet of domain 4, where its side chain makes a salt bridge with Glu792 from an adjacent strand (**S18 Fig**). This interaction is lost in IDE^R847T^, and Glu792 instead bridges to Arg838. Locally the structure is not substantially perturbed from the wild type enzyme as a result of the mutation.

The reduction in V_max_ for the K898 mutant (site 2) is not as marked, but as with the site 1 mutant, the Hill coefficient shows little or no substrate cooperativity. Here, changes in domain 2 are less extensive (strand β11 of the central β-sheet and helices 11, 18) than for IDE^R431A^, but changes in the interface with domain 1 (β6, α5) may account for the reduction in the Hill coefficient. Lys898 makes a salt bridge across the half domain interface with Asp84 from domain 1 in the wild type enzyme (**S19 Fig**). Loss of this interaction in IDE^K898A^, results in a change in conformation of the loop containing residue 898 and a shift of the subsequent helix α32.

Finally, the IDE^R429S^ mutant, which has all three proposed ATP binding sites altered, shows only a small decrease in V_max_ and, in addition, retains substrate cooperativity. Again, changes from the IDE^wt^ simulation are less extensive (RMSD 2.5 Å) than for the IDE^R431A^ mutant, and the interface between domains 1 and 2 is not affected, consistent with the observed Hill coefficient. Arg429 in the wild type enzyme extends into solvent in the absence of bound ATP, and the altered Ser429 side chain interacts with the main chain of Gly432 near the N terminus of helix α15, possibly adding to its stability (**S20 Fig**). This helix shifts in IDE^R429S^ relative to IDE^wt^ in the simulations as a result of this change. In general, it is interesting to note that changes in the polyanion binding surface can lead to varied changes in other parts of the molecule, including the distal activation site in domain 2. This observation is consistent with our earlier observation of crosstalk between the two activation sites [14,16].

## Discussion

Our published structure of ATP bound to IDE [16] demonstrated a binding site located within the positively charged inner surface formed by IDE domains 3 and 4. We were able to demonstrate that mutation of residues that were implicated by the structure as interacting with ATP did in fact reduce activation by ATP, but mutational analysis suggested that ATP could also bind in at least one additional mode. Indeed, the finding of a rather large electropositive surface in the polyanion-binding region is consistent with the existence of a number of potential sites for polyanion binding.

Our molecular dynamics simulations presented here revealed two additional possible modes of ATP binding in this region, and these results are supported by mutagenesis and hydrogen-deuterium exchange data. Mutational analysis also revealed that changes in the polyanion binding site can decrease basal activity and eliminate or reduce substrate cooperativity. These affects appear to result from conformational changes transmitted to other regions of the enzyme directly involved in substrate binding and catalysis.

**Fig 7:**
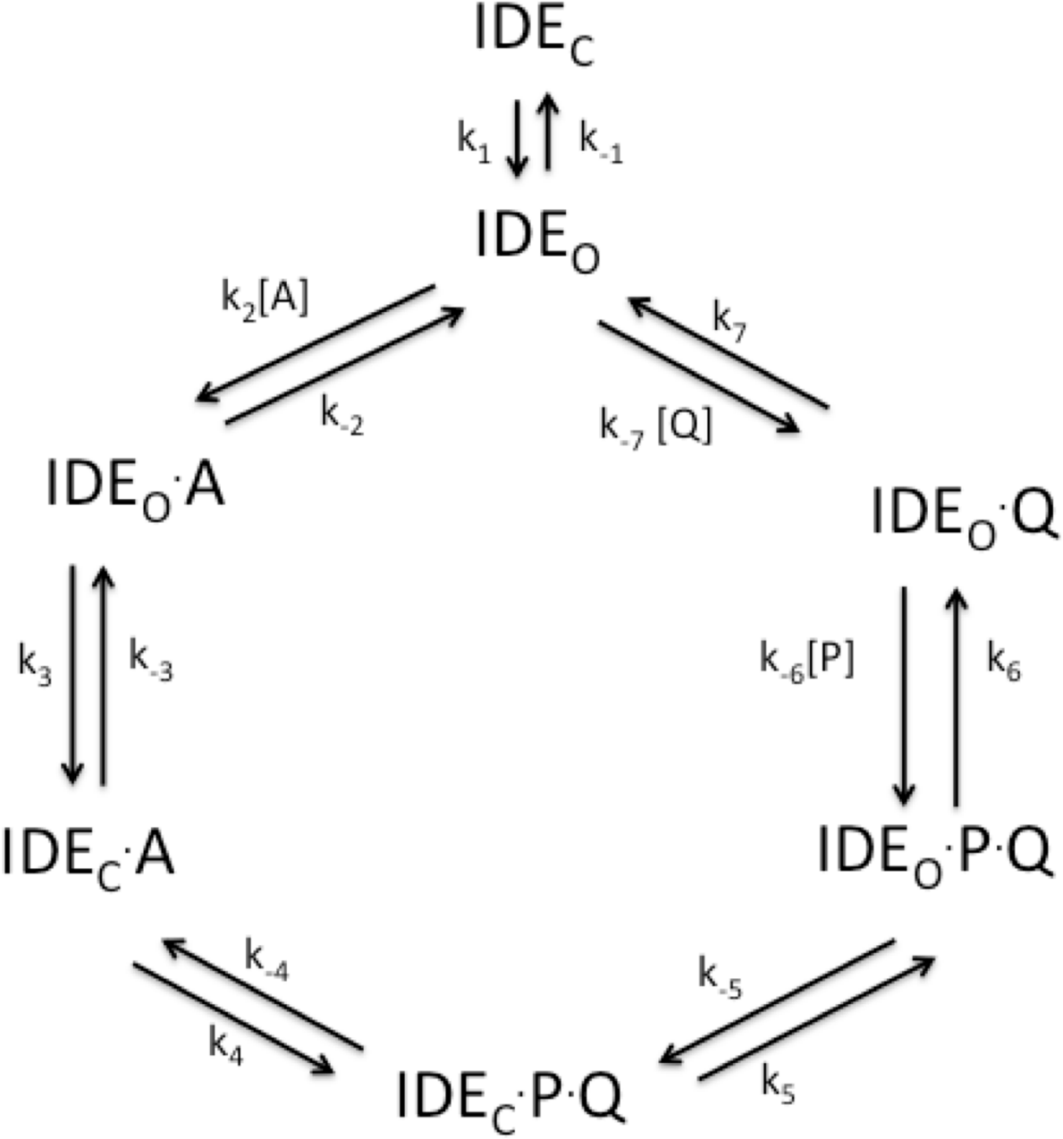
Kinetic scheme for the IDE reaction. IDE_C_ and IDE_O_ represent the closed and open conformations of IDE respectfully. A is a peptide substrate while P and Q are the peptide products derived from the cleavage of A.

Shown in **Fig 7** is the minimal kinetic scheme for the IDE reaction. In this scheme substrate free IDE exists in a closed conformation (IDE_C_) in equilibrium with its open conformation (IDE_O_). Only IDE_O_ is capable of binding substrate, which is then converted to substrate bound enzyme in the closed conformation. The bound substrate is converted to products in the closed conformation, with product release occurring from the open conformation. Studies from Tang and his colleagues (13) showed that the reaction rate is increased when the enzyme conformational equilibrium is shifted toward the open conformation. This indicates that the conversion of the closed conformation to the open conformation contributes to the rate-limiting step.

At high substrate concentrations all enzyme is bound with substrate or product, so the conversion of free IDE_C_ to IDE_O_ cannot contribute to limiting the maximal rate (V_max_ or k_cat_). However, the rate of conversion of IDE_C_^.^P^.^Q to IDE_O_^.^P^.^Q (k_5_ in the forward direction) likely does control the rate of the reaction. In addition, it is known that different IDE substrates are cleaved at different rates. For example glucagon is cleaved twice as fast as Aβ_1–40_, over a hundred fold faster than insulin, and over 500 fold faster than angiotensin (Song et al., unpublished results). This finding requires that a substrate dependent step must contribute to the rate-determining step of the reaction. If this were not the case then all substrates would react at the same maximal rate. Thus k_4_ likely contributes to the rate-determining step of the reaction and the nature of the products could influence k_5_. Polyanion induced conformational changes likely increase the rate of the IDE reaction by increasing the rates associated with k_4_, k_5_, or both.

There are several structural features of IDE that could be affected by the observed conformational changes seen in simulations of the ATP bound and mutant enzyme. As noted above, conformational changes that enhance conversion of the closed form of the IDE-product complex to its open form (IDE_C_^.^P^.^Q to IDE_O_^.^P^.^Q in **Fig 7**) would increase the reaction rate. Such changes would likely occur at the interface between the two halves of the IDE molecule and at the hinge region. It is interesting that in fact these types of conformational changes were observed in the MD simulations.

IDE exists primarily as a homodimer, and it appears that substrate binding at one subunit can affect the overall rate of the other subunit [12,35]. Thus conformational changes that affect the interaction between monomeric units within the IDE dimer could also affect the rate of the reaction. In addition the monomeric form of IDE, although retaining ~25% activity toward a small synthetic substrate, is not activated by polyanions nor does it display activation by peptides [33]. Thus changes seen in dimer interface elements with ATP bound at two of the sites in the simulations and in the hydrogen-deuterium exchange studies suggest that changes in the dimer interface play a role in allosteric activation by polyanions.

Also of significance is the distal binding region [13,14], which binds small peptides or the extended portion of large substrates. With peptide substrates that are not large enough to extend to the distal site, small peptides bound at the distal site increase the reaction rate. Thus binding at the distal site appears responsible for the allosteric kinetics observed with small substrates. Mutations in this distal site not only affect the allosteric nature of the enzyme, but decrease the ability of polyanions to activate [14]. Similarly mutations that affect the polyanion site also decrease activation by small peptides [16] showing a linkage between the polyanion binding site and the distal allosteric site. Such a linkage is best explained by conformation changes transmitted between these sites. In addition, we found that mutation of Cys904 and Asn575, which likely form a hydrogen bond pair, reduced both activity and polyanion activation [32]. Since these residues are far from the distal and polyanion-binding region, such affects can only be explained by conformational changes produced when this hydrogen bonded pair is disrupted by mutation. The dynamics simulations of polyanion binding region mutants reported here show conformational changes transmitted to the distal site domain, consistent with the reported linkage between the two allosteric sites.

## Conclusions

Previous studies have demonstrated the ability of ligands, including peptides and polyanions, to activate IDE. In this work, at least three ATP binding modes on the extended cationic surface of the substrate-binding chamber are identified. The increased rate of substrate cleavage induced by ATP binding, and presumably other polyanions, is suggested to occur through conformational changes that alter the rate of the catalytic step and/or partitioning between open and closed conformations of IDE. Elements at the interface between the two halves of the enzyme were seen to undergo conformational changes in dynamics simulations with ATP bound at any of the three polyanion sites, a possible structural basis for activation. Mutagenesis of the polyanion binding surface shows it to be a critical region of the enzyme where sequence changes produce effects on both basal activity and the capacity for allosteric activation. Changes to this surface likely affect structural elements at the active site and the distal site domain based on dynamics simulations. The degree of polyanion activation for larger polypeptide substrates is not yet fully established. Functionally, however, the presence of multiple polyanion binding modes may be important in retaining activation for substrates that occlude one or two of the polyanion sites, which includes insulin and amylin based on crystal structures [13,36,37]. If so, then molecules that target one binding site over others may be useful for selective modulation of IDE activity. These studies point out the conformational flexibility of IDE and the significant effect on catalysis that conformational changes can have with this enzyme.

## Supporting Information

S1 Fig RMSD of the IDE^wt^ and IDE mutants simulation trajectories versus time. (TIF)

S2 Fig RMSD of ATP bound at each of three different sites simulation trajectories versus time. (TIF)

S3 Fig Superposition snapshots of IDE with ATP bound at Site 1 at 5, 10, 15, 20, 25, and 30 ns time points of the simulations. (TIF)

S4 Fig Superposition snapshots of IDE with ATP bound at Site 2 at 5, 10, 15, 20, 25, and 30 ns time points of the simulations. (TIF)

S5 Fig Superposition snapshots of IDE with ATP bound at Site 3 at 5, 10, 15, 20, 25, and 30 ns time points of the simulations. (TIF)

S6 Fig Secondary structure assignment of IDE^wt^ for each residue versus time in the simulation. (TIF)

S7 Fig Secondary structure assignment of IDE^wt^ with ATP bound at Site 1 for each residue versus time in the simulation. (TIF)

S8 Fig Secondary structure assignment of IDE^wt^ with ATP bound at Site 2 for each residue versus time in the simulation. (TIF)

S9 Fig Secondary structure assignment of IDE^wt^ with ATP bound at Site 3 for each residue versus time in the simulation. (TIF)

S10 Fig Secondary structure assignment of IDE^R429S^ for each residue versus time in the simulation. (TIF)

S11 Fig Secondary structure assignment of IDE^R431A^ for each residue versus time in the simulation. (TIF)

S12 Fig Secondary structure assignment of IDE^R847T^ for each residue versus time in the simulation. (TIF)

S13 Fig Secondary structure assignment of IDE^R898A^ for each residue versus time in the simulation. (TIF)

S14 Fig Structural changes accompanying ATP binding at Site 1. ATP bound to IDE at Site 1 is shown in a stick figure representation with green carbons. Superimposed unliganded IDE (cyan) and IDE-ATP (gray) are shown as cartoons with key interacting side chains as stick representations.
(TIF)

S15 Fig Structural changes accompanying ATP binding at Site 2. ATP bound to IDE at Site 2 is shown in a stick figure representation with green carbons. Superimposed unliganded IDE (cyan) and IDE-ATP (gray) are shown as cartoons with key interacting side chains as stick representations.
(TIF)

S16 Fig Structural changes accompanying ATP binding at Site 3. ATP bound to IDE at Site 3 is shown in a stick figure representation with green carbons. Superimposed unliganded IDE (cyan) and IDE-ATP (gray) are shown as cartoons with key interacting side chains as stick representations.
(TIF)

S17 Fig Structural changes in IDE^R431A^ versus the wild type enzyme. Superimposed IDE^wt^ (cyan) and IDE^R431A^ (gray) are shown as cartoons with key side chains at the mutation site shown as stick representations.
(TIF)

S18 Fig Structural changes in IDE^R847T^ versus the wild type enzyme. Superimposed IDE^wt^ (cyan) and IDE^R847T^ (gray) are shown as cartoons with key side chains at the mutation site shown as stick representations.
(TIF)

S19 Fig Structural changes in IDE^K898A^ versus the wild type enzyme. Superimposed IDE^wt^ (cyan) and IDE^K898A^ (gray) are shown as cartoons with key side chains at the mutation site shown as stick representations.
(TIF)

S20 Fig Structural changes in IDE^R429S^ versus the wild type enzyme. Superimposed IDE^wt^ (cyan) and IDE^R429A^ (gray) are shown as cartoons with key side chains at the mutation site shown as stick representations.
(TIF)
