# Supplementary figures and images for: An Extended Polyanion Activation Surface in Insulin Degrading Enzyme
Source: PLoS One. 2015 Jul 17;10(7):e0133114. doi: 10.1371/journal.pone.0133114 (PMC4506039; doi:10.1371/journal.pone.0133114)

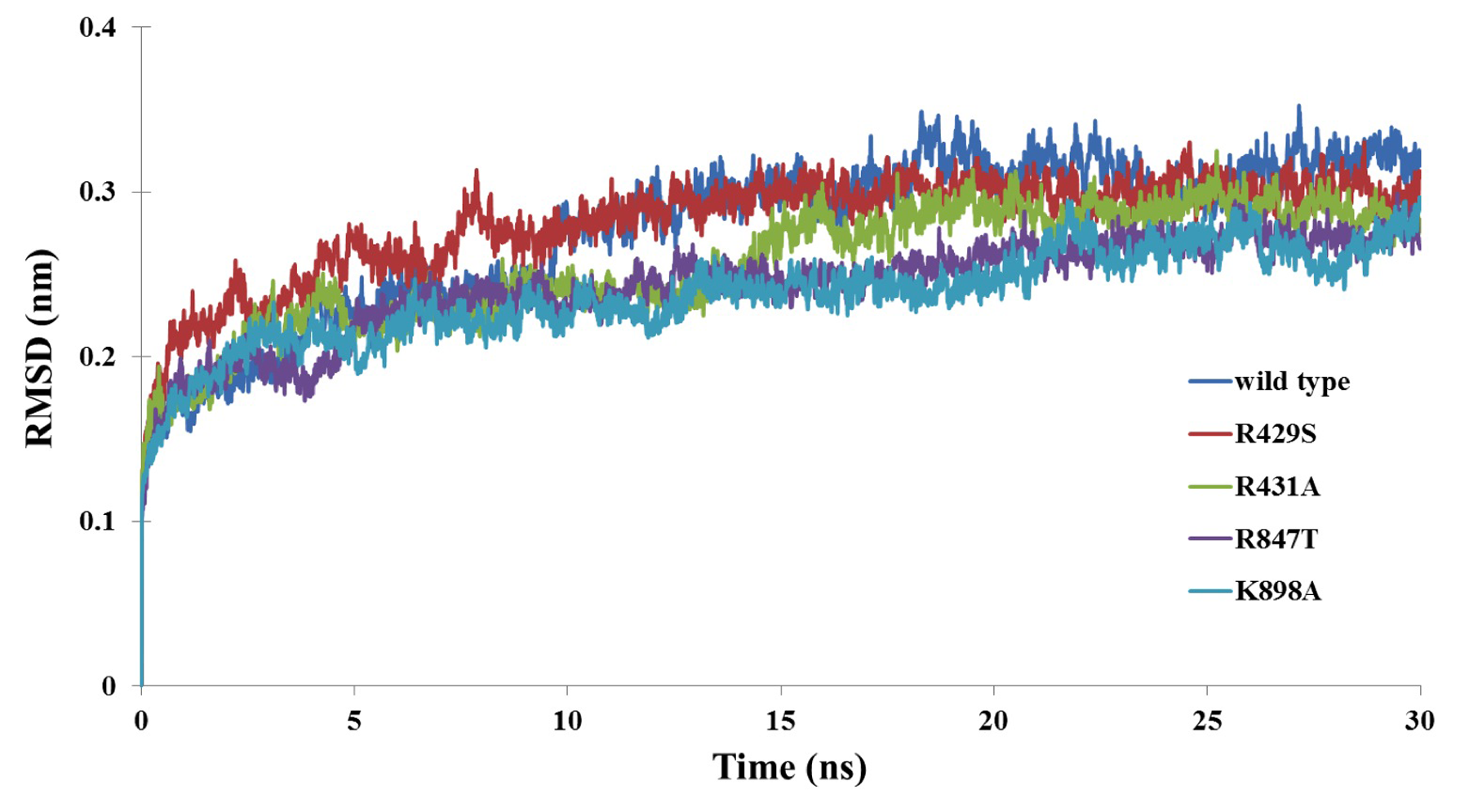

Supplement: S1 Fig — (TIF) [file pone.0133114.s001.tif]

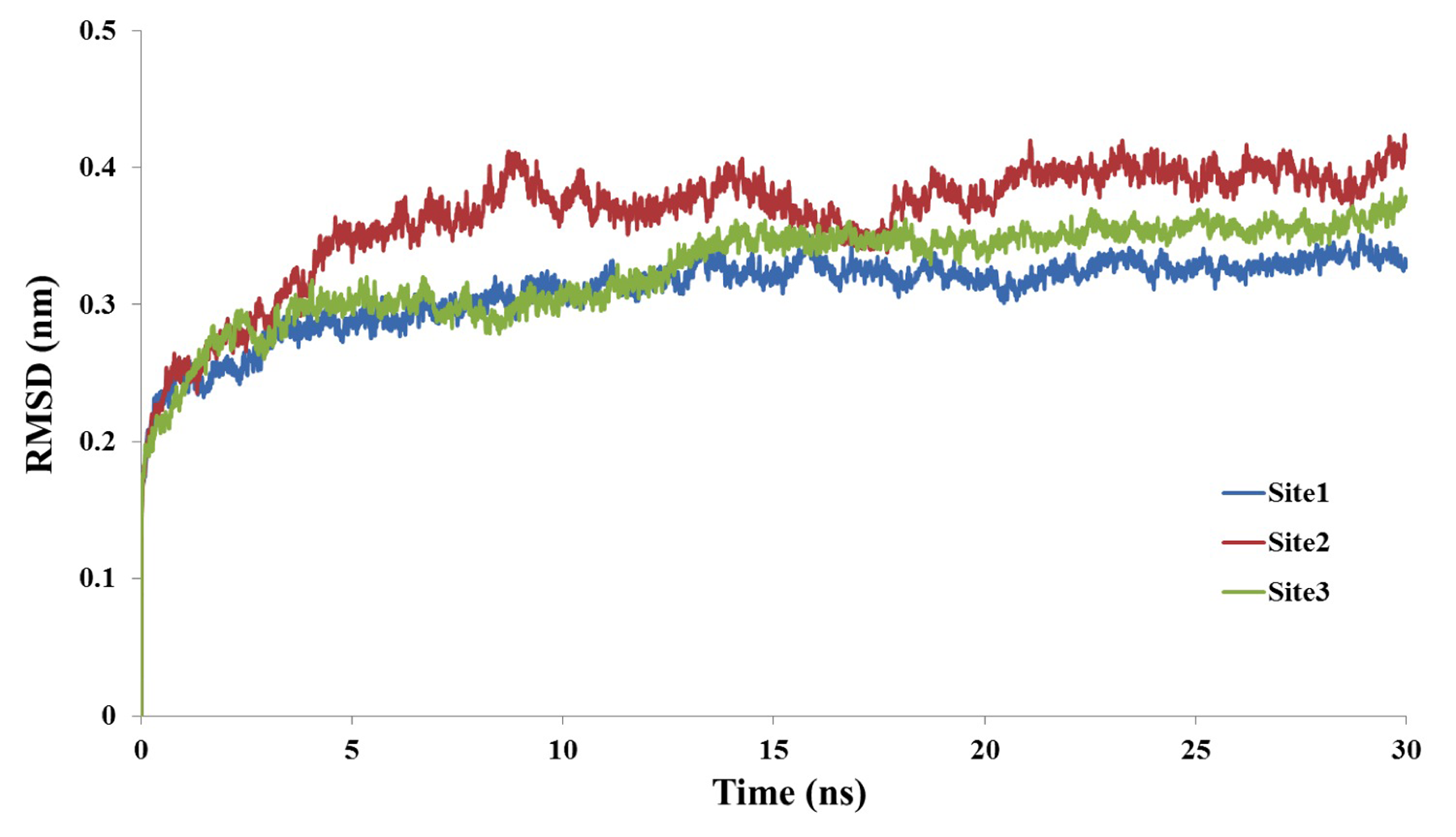

Supplement: S2 Fig — (TIF) [file pone.0133114.s002.tif]

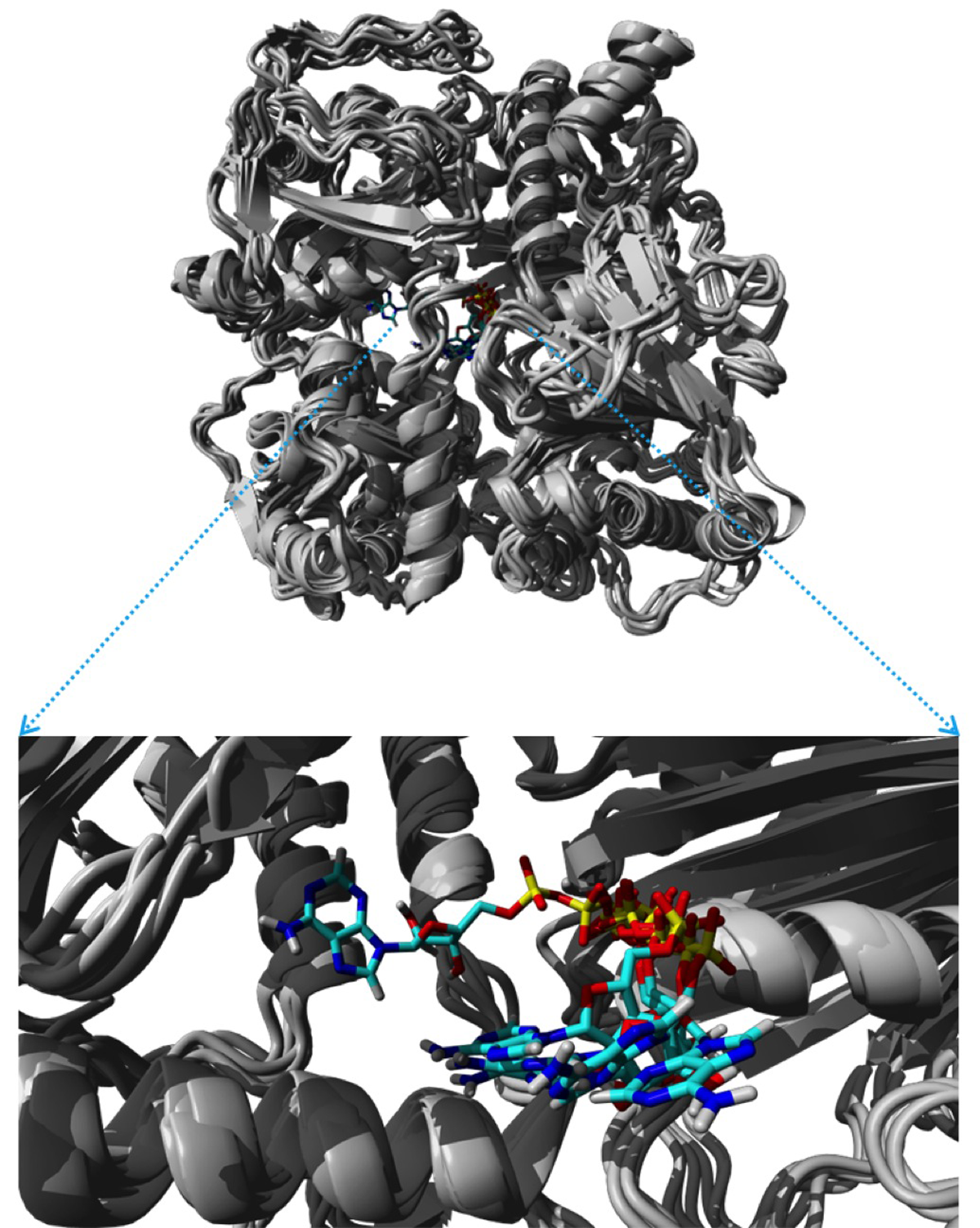

Supplement: S3 Fig — (TIF) [file pone.0133114.s003.tif]

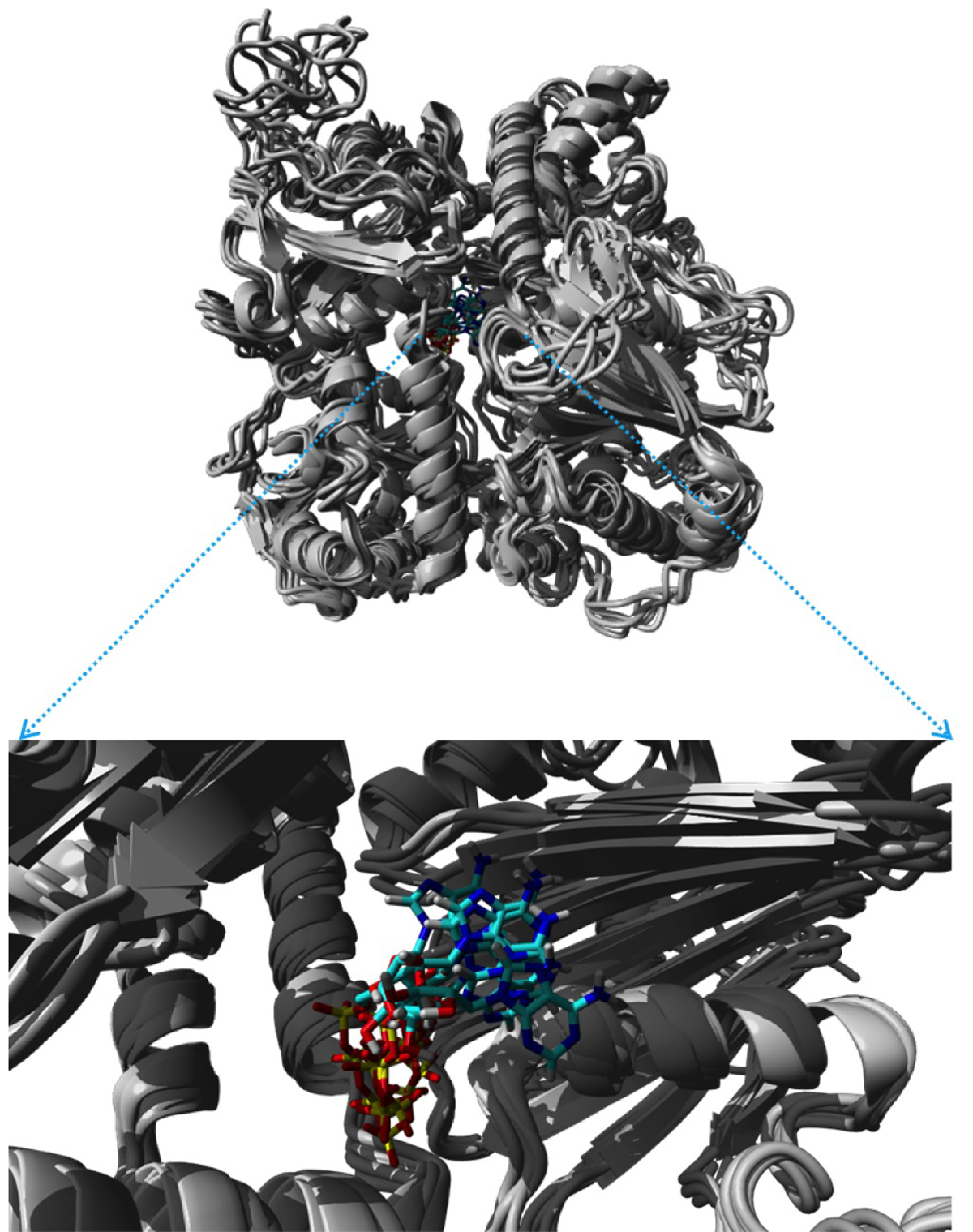

Supplement: S4 Fig — (TIF) [file pone.0133114.s004.tif]

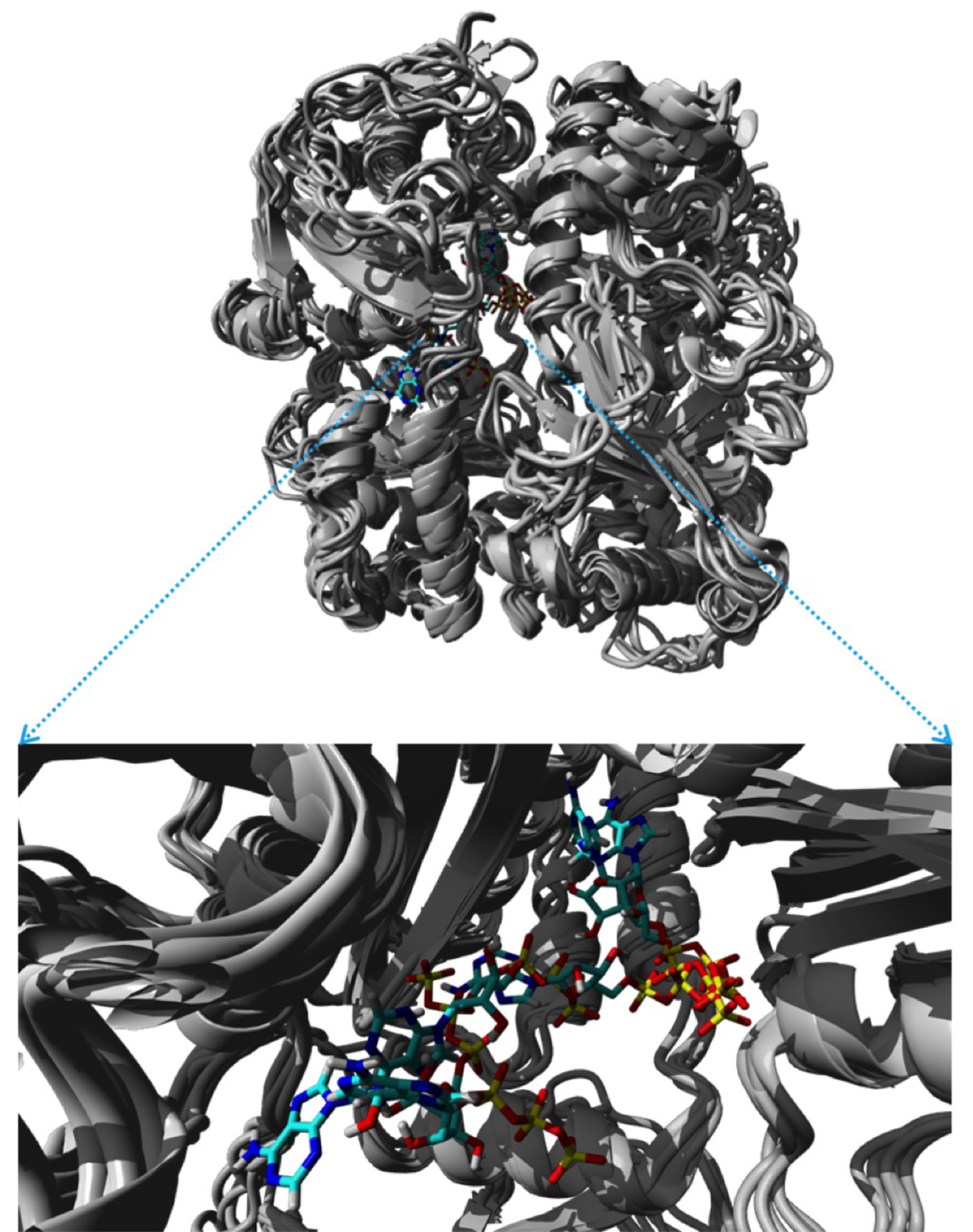

Supplement: S5 Fig — (TIF) [file pone.0133114.s005.tif]

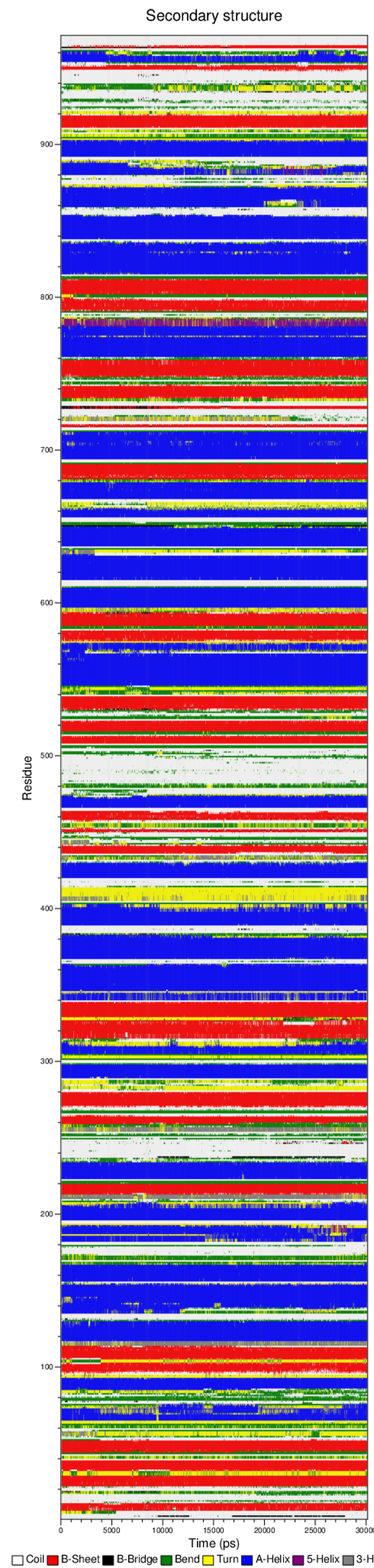

Supplement: S6 Fig — (TIF) [file pone.0133114.s006.tif]

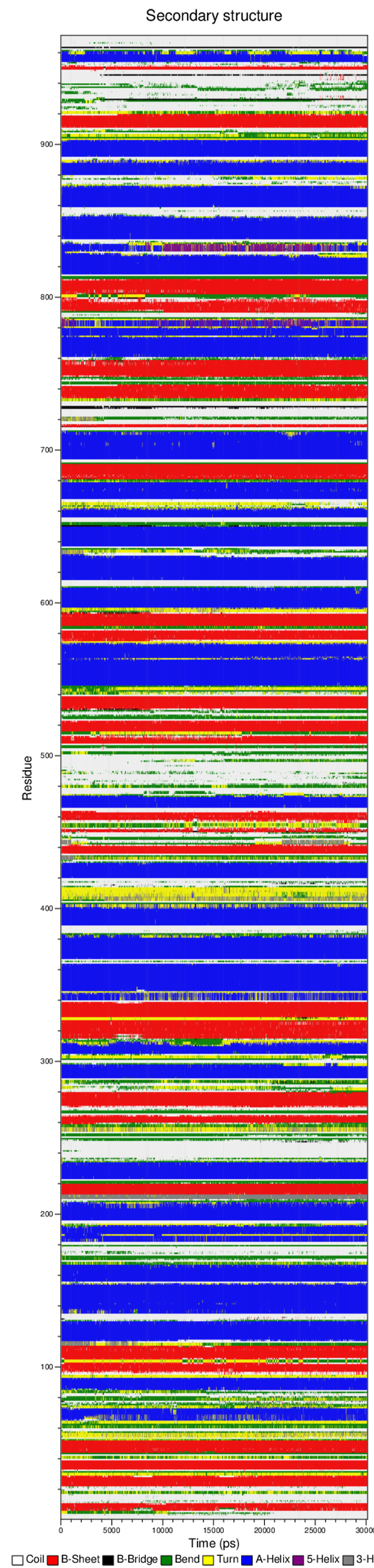

Supplement: S7 Fig — (TIF) [file pone.0133114.s007.tif]

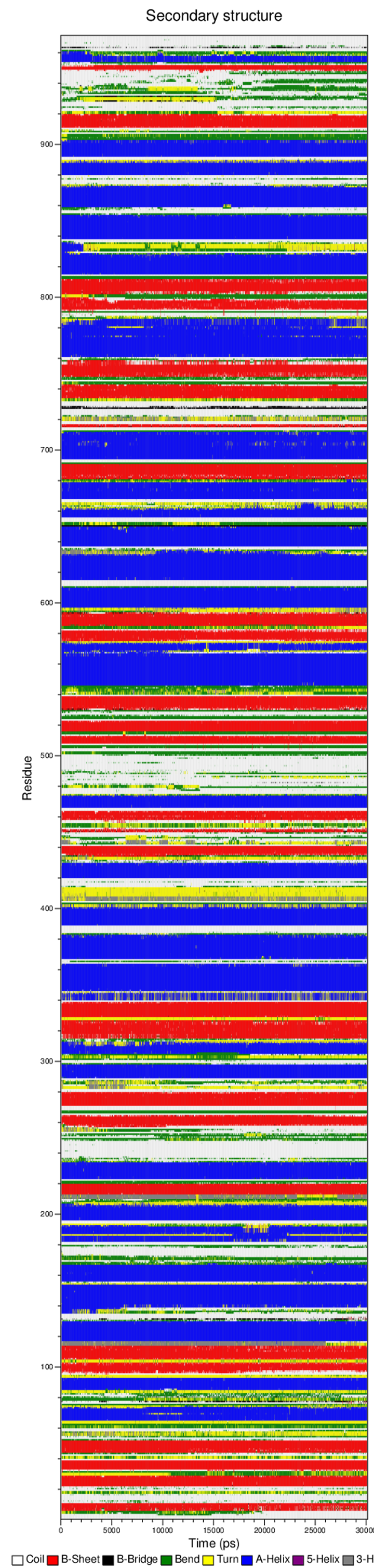

Supplement: S8 Fig — (TIF) [file pone.0133114.s008.tif]

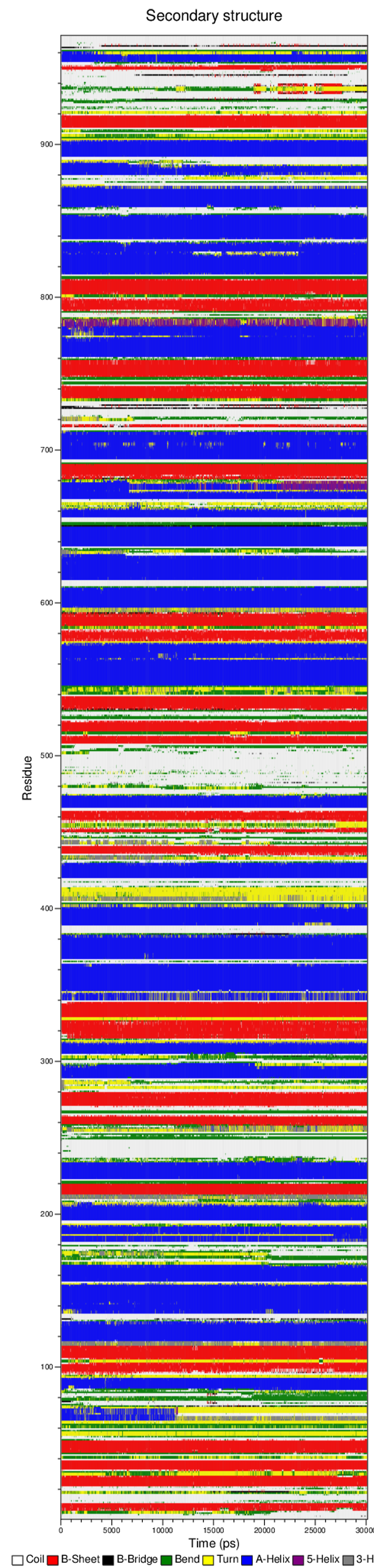

Supplement: S9 Fig — (TIF) [file pone.0133114.s009.tif]

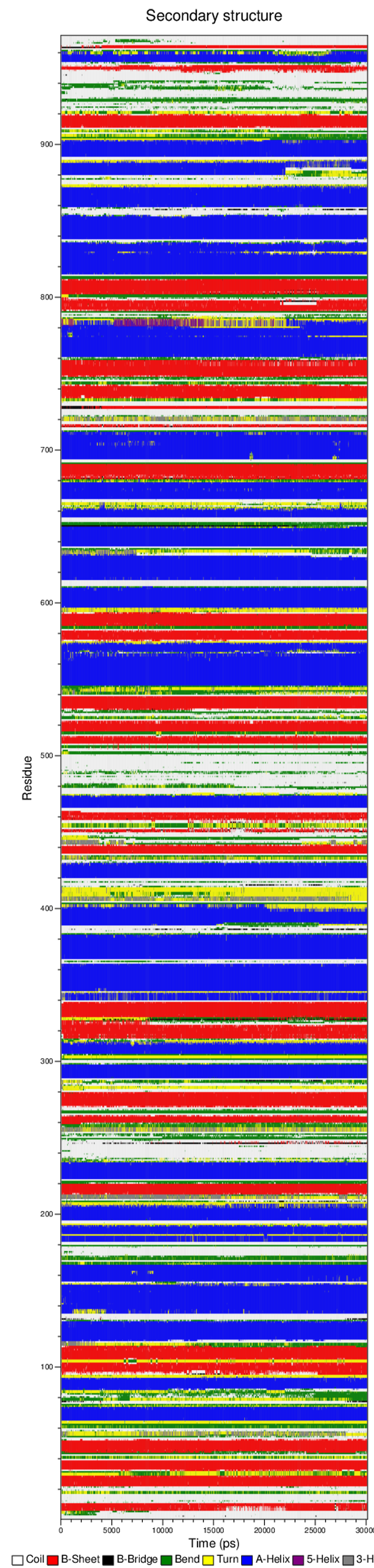

Supplement: S10 Fig — (TIF) [file pone.0133114.s010.tif]

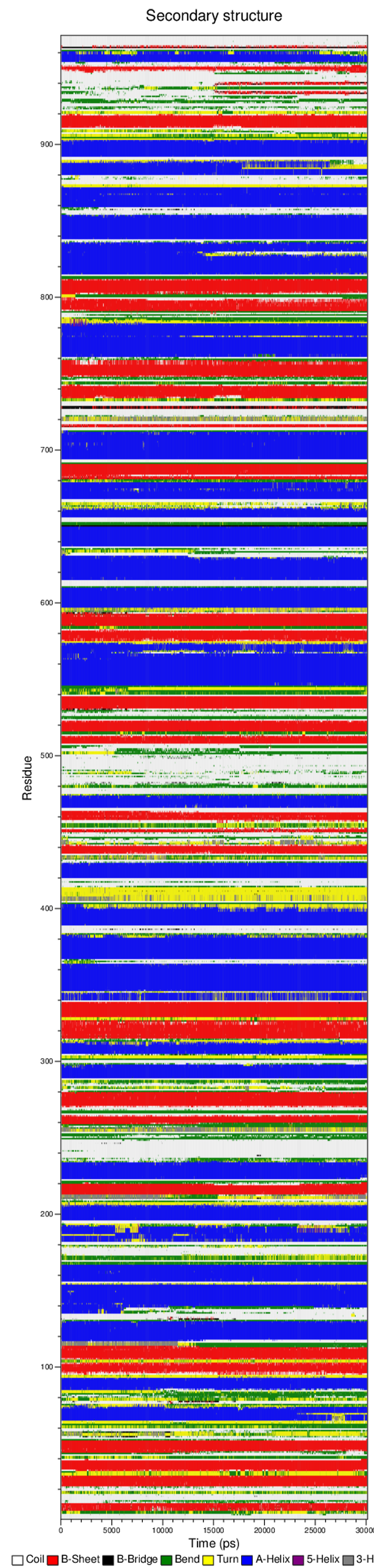

Supplement: S11 Fig — (TIF) [file pone.0133114.s011.tif]

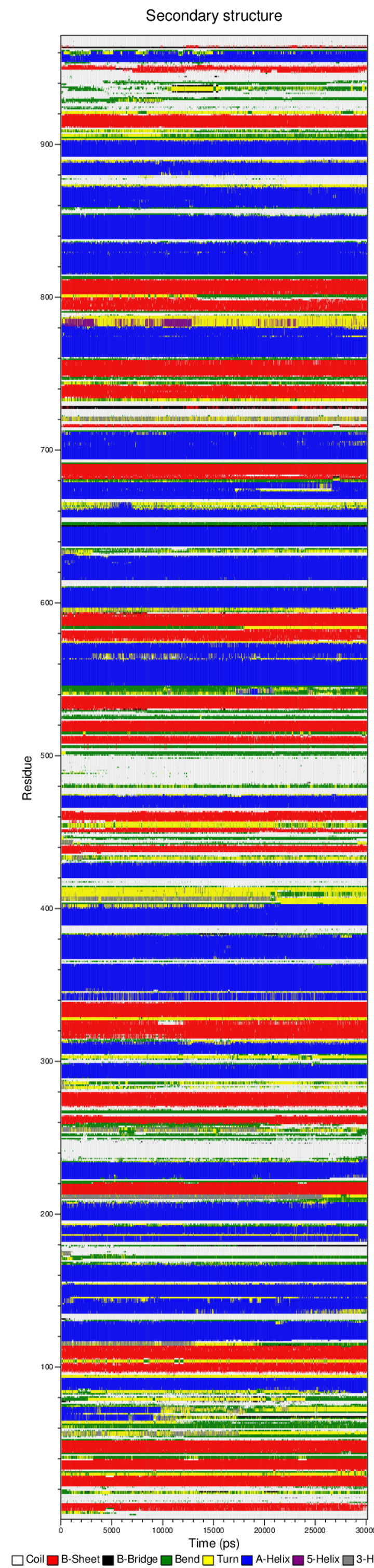

Supplement: S12 Fig — (TIF) [file pone.0133114.s012.tif]

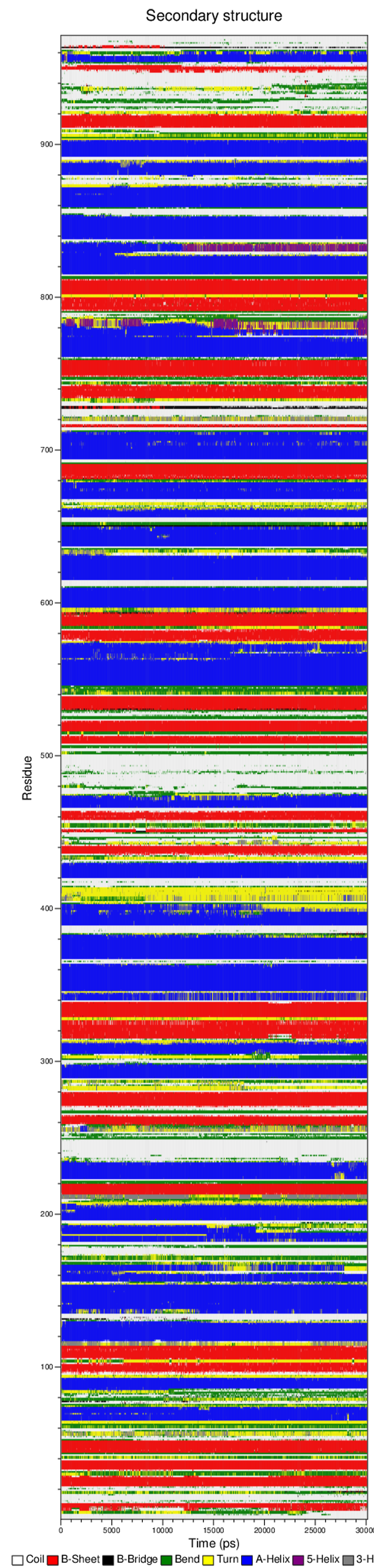

Supplement: S13 Fig — (TIF) [file pone.0133114.s013.tif]

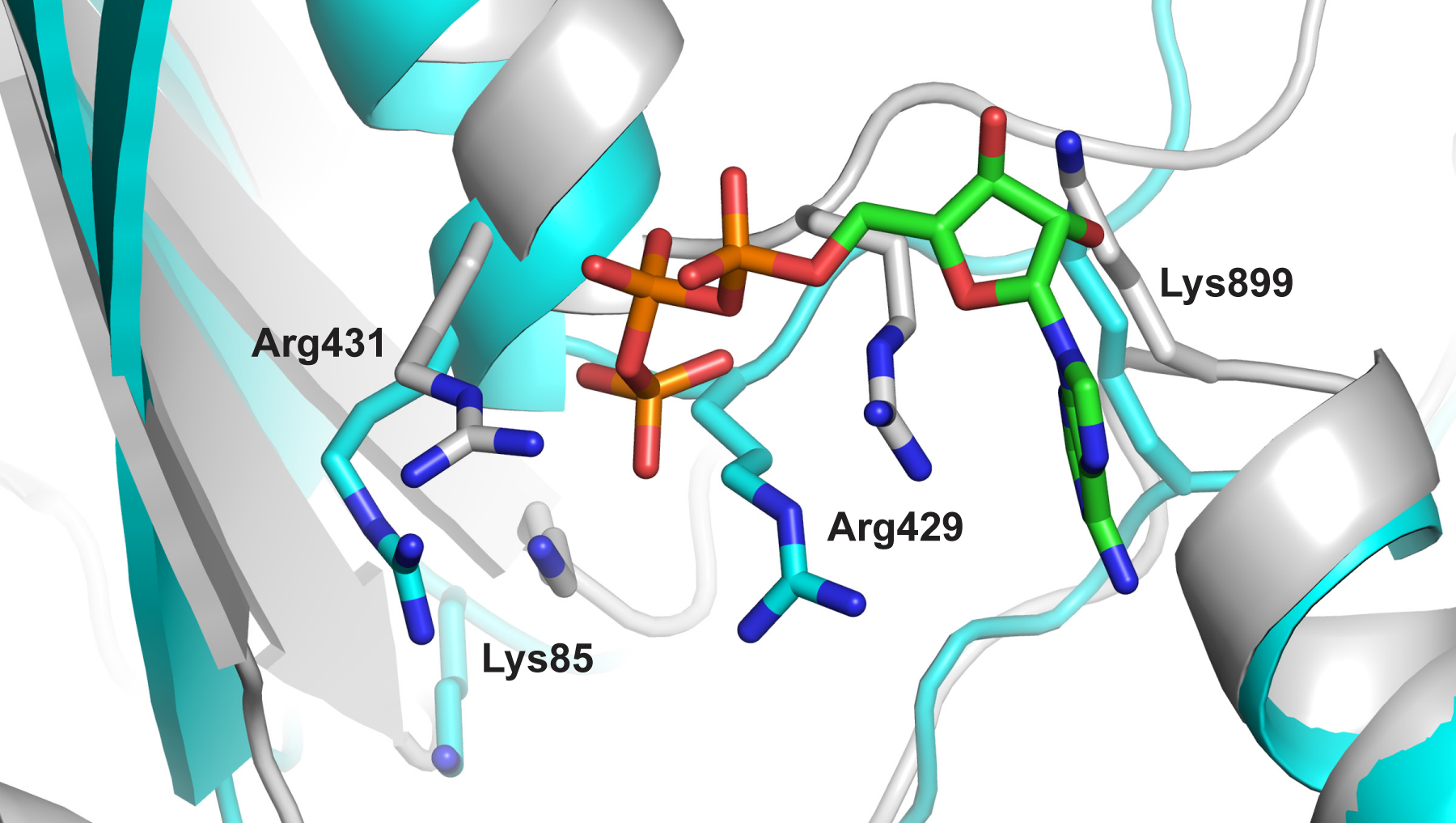

Supplement: S14 Fig — ATP bound to IDE at Site 1 is shown in a stick figure representation with green carbons. Superimposed unliganded IDE (cyan) and IDE-ATP (gray) are shown as cartoons with key interacting side chains as stick representations. (TIF) [file pone.0133114.s014.tif]

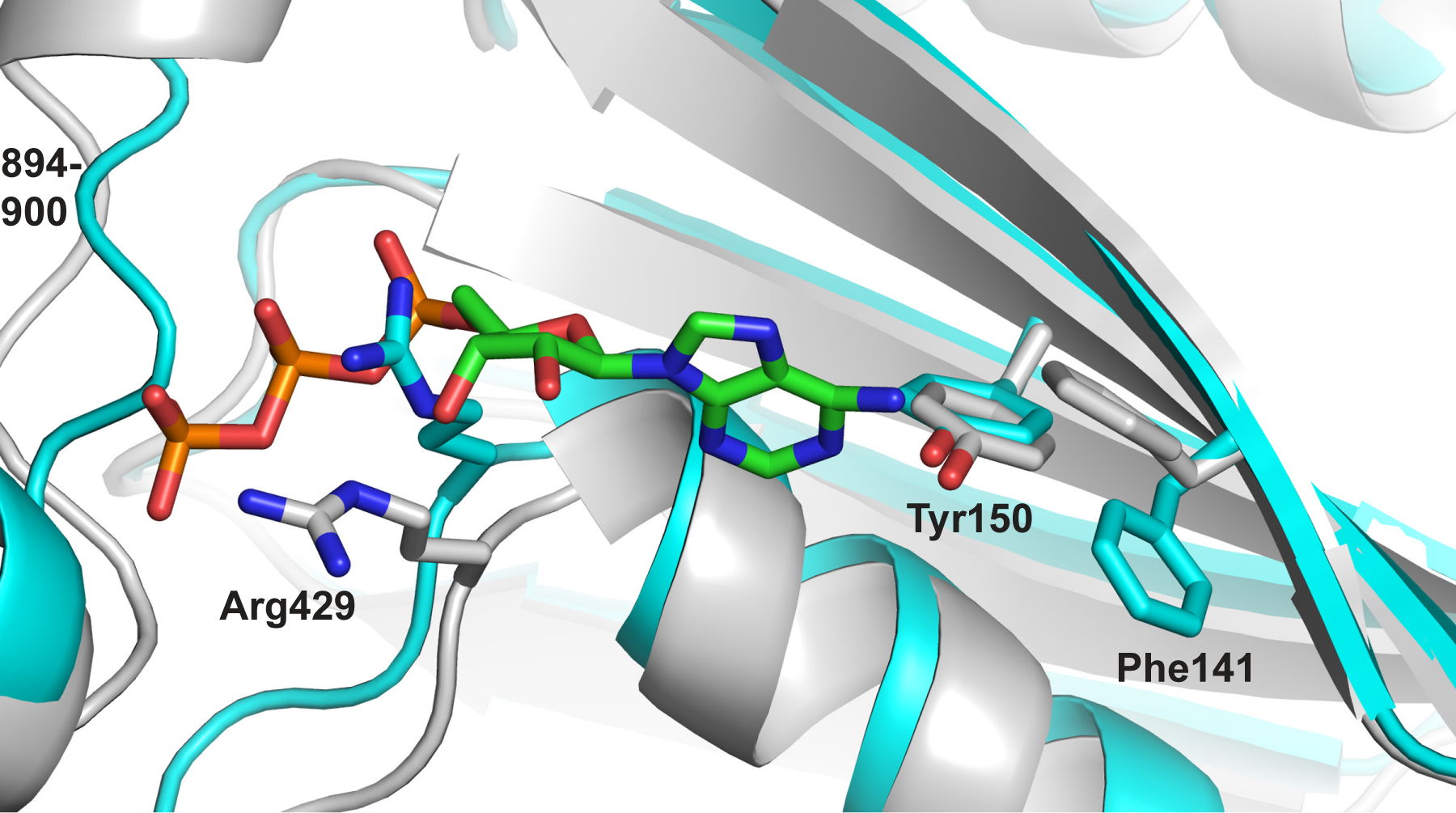

Supplement: S15 Fig — ATP bound to IDE at Site 2 is shown in a stick figure representation with green carbons. Superimposed unliganded IDE (cyan) and IDE-ATP (gray) are shown as cartoons with key interacting side chains as stick representations. (TIF) [file pone.0133114.s015.tif]

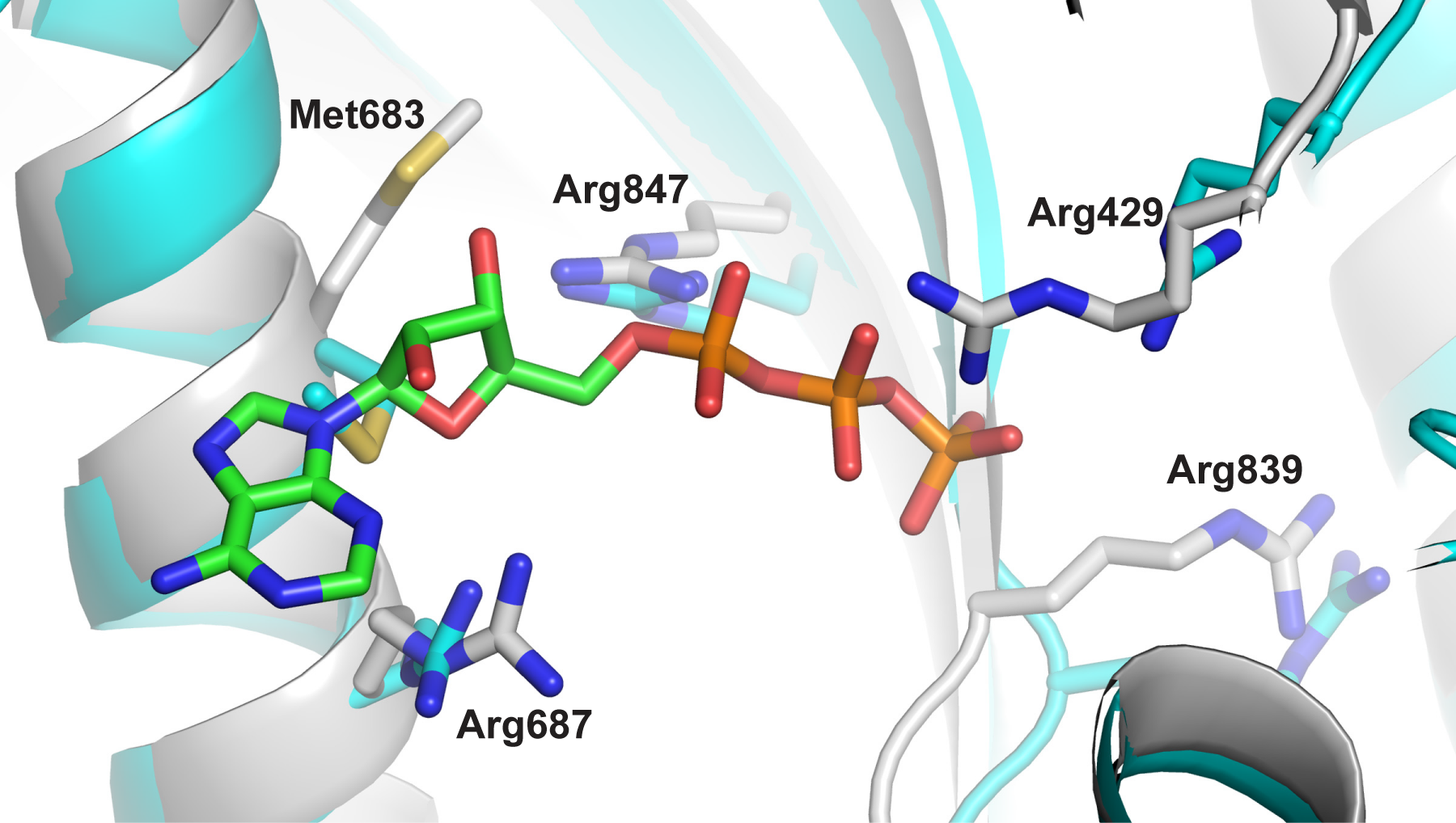

Supplement: S16 Fig — ATP bound to IDE at Site 3 is shown in a stick figure representation with green carbons. Superimposed unliganded IDE (cyan) and IDE-ATP (gray) are shown as cartoons with key interacting side chains as stick representations. (TIF) [file pone.0133114.s016.tif]

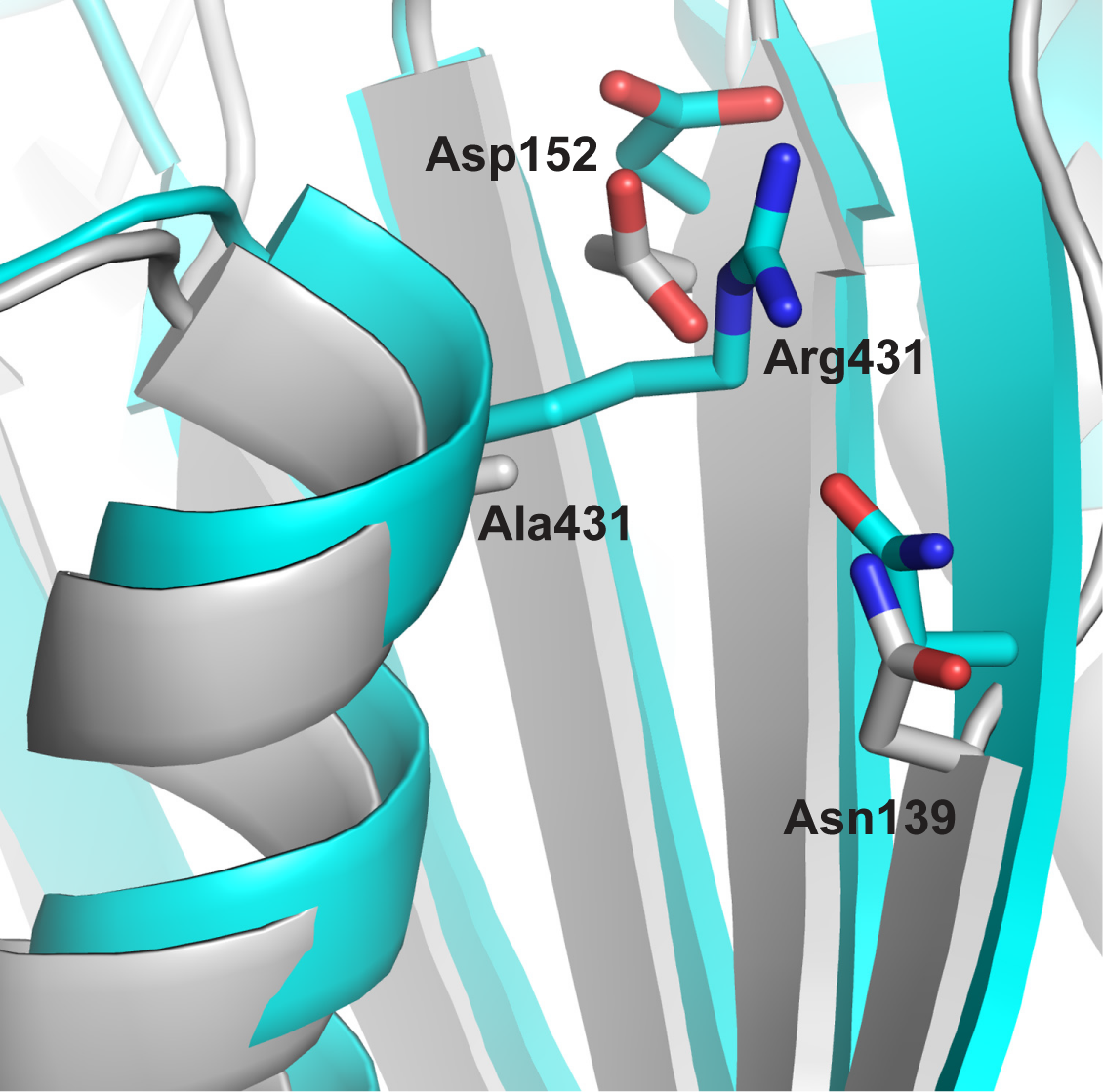

Supplement: S17 Fig — Superimposed IDEwt (cyan) and IDER431A (gray) are shown as cartoons with key side chains at the mutation site shown as stick representations. (TIF) [file pone.0133114.s017.tif]

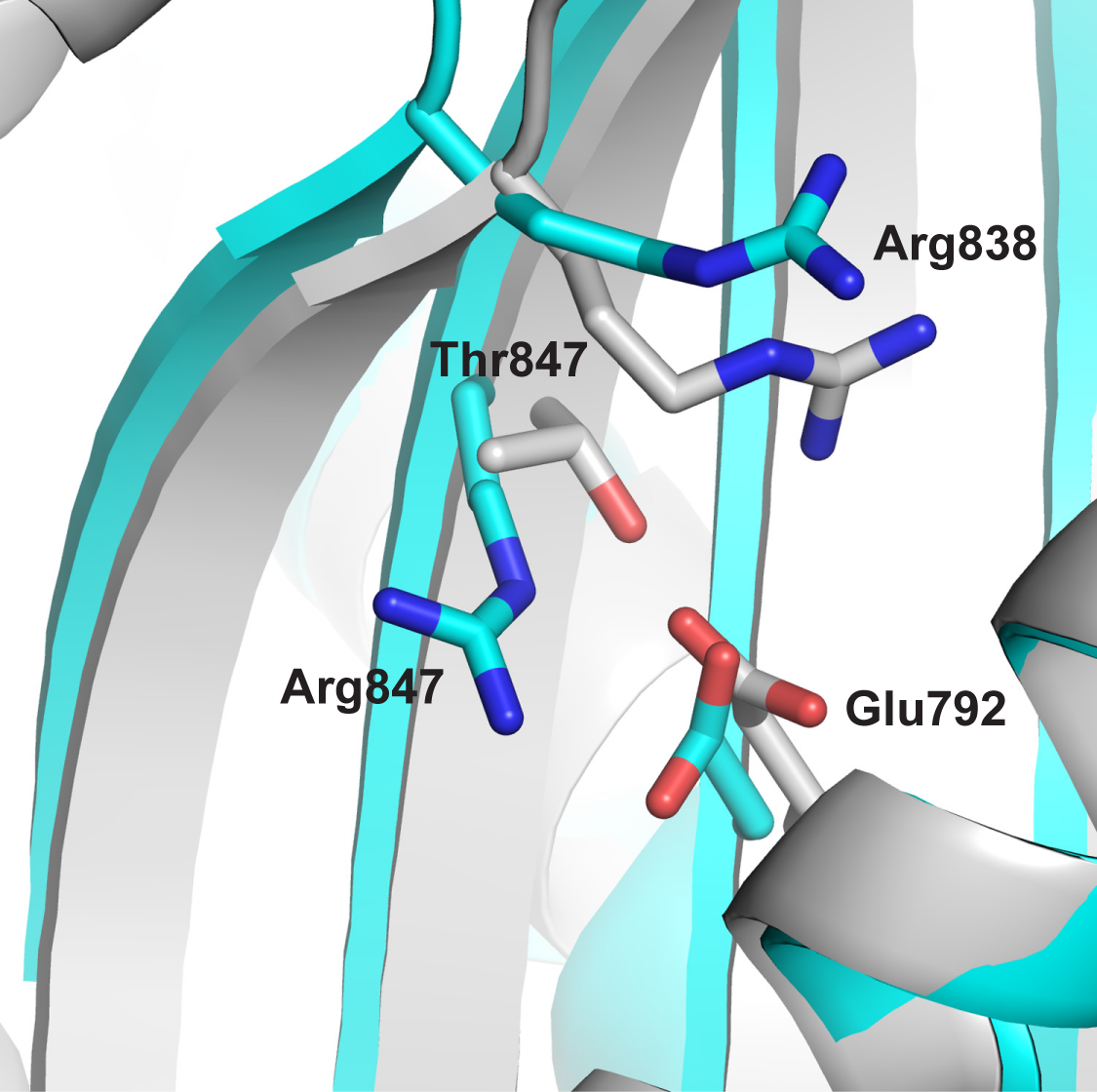

Supplement: S18 Fig — Superimposed IDEwt (cyan) and IDER847T (gray) are shown as cartoons with key side chains at the mutation site shown as stick representations. (TIF) [file pone.0133114.s018.tif]

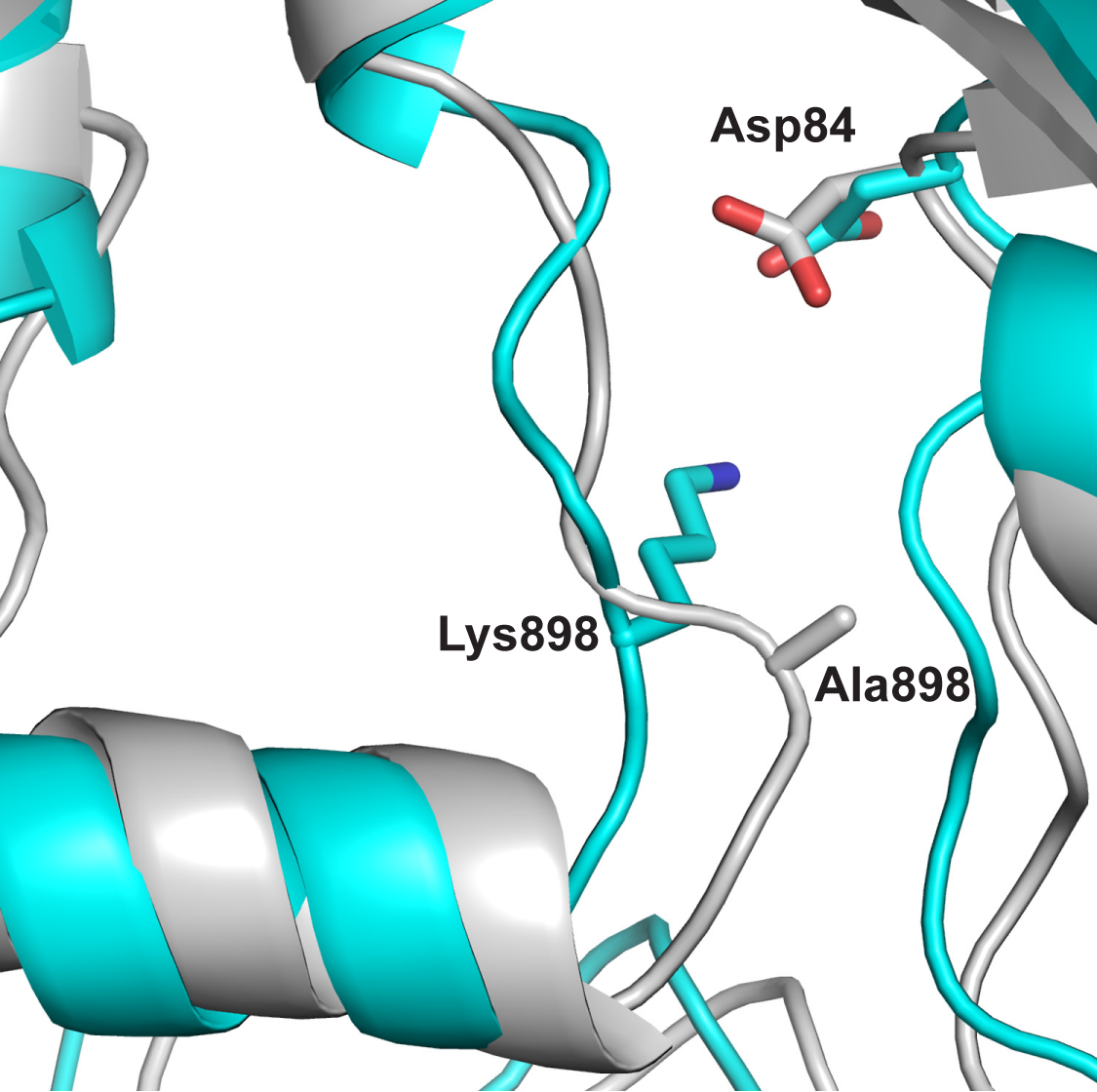

Supplement: S19 Fig — Superimposed IDEwt (cyan) and IDEK898A (gray) are shown as cartoons with key side chains at the mutation site shown as stick representations. (TIF) [file pone.0133114.s019.tif]

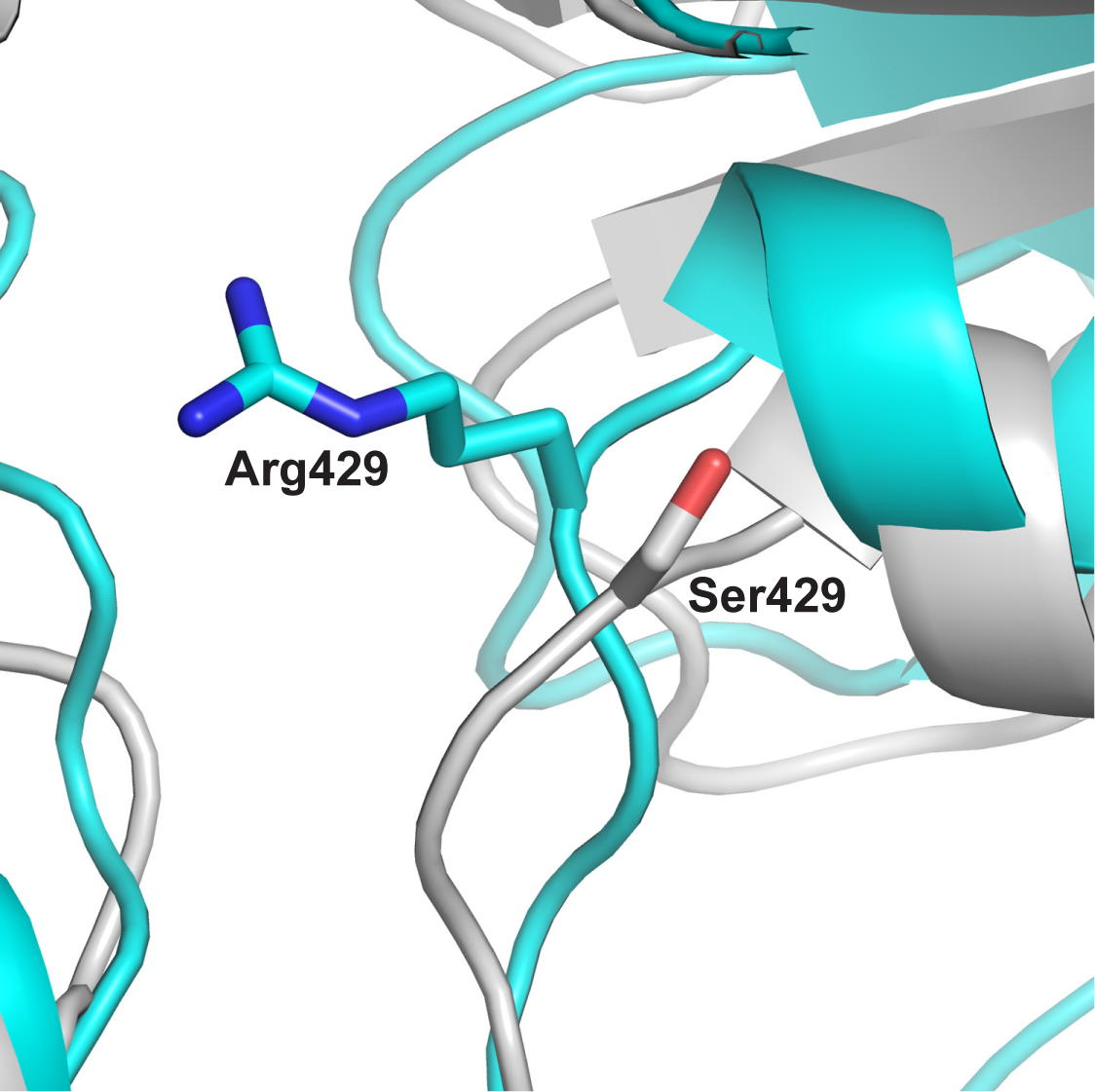

Supplement: S20 Fig — Superimposed IDEwt (cyan) and IDER429A (gray) are shown as cartoons with key side chains at the mutation site shown as stick representations. (TIF) [file pone.0133114.s020.tif]
